# Unveiling Salt Tolerance Mechanisms and Hub Genes in Alfalfa (*Medicago sativa* L.) Through Transcriptomic and WGCNA Analysis

**DOI:** 10.3390/plants13223141

**Published:** 2024-11-08

**Authors:** Fengdan Wang, Hanfu Wu, Mei Yang, Wen Xu, Wenjie Zhao, Rui Qiu, Ning Kang, Guowen Cui

**Affiliations:** 1 Department of Grassland Science, College of Animal Science and Technology, Northeast Agricultural University, Harbin 150030, China; wangfengdan122@163.com (F.W.); w995450205@sina.com (H.W.); yangmei199611@163.com (M.Y.); xw20220921@163.com (W.X.); 13310467886@163.com (W.Z.); qr020327@163.com (R.Q.); 2 Department of Animal Science, College of Animal Science and Technology, Inner Mongolia Agricultural University, Hohhot 010018, China; 13789734560@163.com

**Keywords:** alfalfa, salt stress, transcriptomic analysis, WGCNA, hub gene

## Abstract

Alfalfa (*Medicago sativa* L.), an important forage crop with high nutritional value and good palatability, plays a vital role in the development of animal husbandry in China. In Northeast China, there are vast areas of saline–alkali land that remain undeveloped. Given that alfalfa is a highly adaptable forage crop, exploring its salt tolerance at the molecular transcriptional level and identifying salt-tolerant genes has great significance for breeding salt-resistant alfalfa varieties. This also provides valuable genetic resources for better utilization of saline–alkali land. In this study, we conducted two rounds of screening on 41 alfalfa varieties and identified WL168 as a salt-sensitive variety and Longmu801 as a salt-tolerant variety. After 7 days of 300 mM salt stress, both varieties showed a decreasing trend in plant height, fresh weight, and dry weight over time, but Longmu801 demonstrated better water retention ability compared to WL168. Chlorophyll content also declined, but chlorophyll a and total chlorophyll levels in Longmu801 were higher than in WL168. Hydrogen peroxide and malondialdehyde levels increased overall, but Longmu801 had significantly lower levels than WL168 under prolonged stress. Both varieties showed increasing trends in soluble sugars, proline, and antioxidant enzymes (SOD, POD, CAT), with Longmu801 significantly outperforming WL168. This suggests that the two varieties share similar growth and physiological response mechanisms, with their differences primarily arising from variations in indicator levels. In the above, comparisons between varieties were conducted based on the relative values of the indicators in relation to their controls. Transcriptomic analysis revealed that under salt stress, Longmu801 had 16,485 differentially expressed genes (DEGs) relative to its control, while WL168 had 18,726 DEGs compared to its control. Among these, 2164 DEGs shared the same expression trend, with GO functions enriched in response to oxidative stress, nucleus, plasma membrane, and others. The KEGG pathways were enriched in phenylpropanoid biosynthesis, protein processing in the endoplasmic reticulum, starch and sucrose metabolism, and others. This suggests that alfalfa’s transcriptional response mechanism to salt stress involves these pathways. Additionally, the variety-specific DEGs were also enriched in the same KEGG pathways and GO functions, indicating that the differences between the two varieties stem from their unique stress-responsive DEGs, while their overall mechanisms for coping with stress remain similar. To further identify salt stress-related genes, this study conducted WGCNA analysis using 32,683 genes and physiological indicators. Six modules closely related to physiological traits were identified, and the top five genes ranked by degree in each module were selected as hub genes. Further analysis of these hub genes identified five genes directly related to salt stress: *Msa085011*, *Msa0605650*, *Msa0397400*, *Msa1258740*, and *Msa0958830*. Mantel test analysis revealed that these genes showed strong correlations with physiological indicators. This study will provide important insights for breeding salt-tolerant alfalfa varieties.

## 1. Introduction

Alfalfa (*Medicago sativa* L.) is an important perennial legume forage crop that is widely cultivated globally, with a planting area of approximately 45 million hectares. The history of its use as forage for feeding livestock dates back over 3300 years [1,2]. Alfalfa is known as the “king of forages” due to its high crude protein content, good palatability, and high biomass yield [3,4]. It also has a well-developed root system and strong adaptability [5], demonstrating characteristics such as cold tolerance [6], drought tolerance [7], and salt tolerance [8]. Additionally, alfalfa roots host nitrogen-fixing rhizobia, which play a role in nitrogen fixation [9], contributing to its ecological value [10]. Due to these superior traits, alfalfa has become the most widely planted forage crop in the world [11].

In China, alfalfa has also been utilized for a long time. As early as 2000 years ago, during the Western Han Dynasty, Zhang Qian introduced alfalfa to China via the Silk Road [12]. With the development of China’s animal husbandry industry and the increasing domestic demand for livestock products, the demand for high-quality alfalfa as forage has steadily increased [13]. However, domestic alfalfa production is far from meeting market demand. The average self-sufficiency rate of high-quality alfalfa in China is only 64% [14]. Between 2012 and 2020, China’s alfalfa hay imports increased from 440,000 tons to 1.36 million tons [15], highlighting a significant gap in alfalfa production. The development of China’s alfalfa industry faces several challenges. For example, the germplasm resources of alfalfa are relatively poor, and modern forage breeding in China began relatively late in the second half of the 20th century, compared to developed countries [16]. Good arable land is typically used for traditional crops, and to alleviate land-use conflicts and prevent competition between grain and forage crops, alfalfa and other forages are often planted on marginal lands, such as those prone to drought or salinity [17,18]. This poses significant challenges for the production of high-quality alfalfa, making it urgent to accelerate alfalfa breeding and obtain salt-tolerant germplasm resources. Molecular breeding techniques provide an effective solution to this challenge [19,20].

Currently, many researchers have improved alfalfa’s stress resistance by identifying stress-related genes and using transgenic molecular breeding methods. Tang et al. heterologously expressed a zinc-finger protein (Cys2/His2-type zinc-finger protein) from Glycine soja in alfalfa and found that the transgenic lines exhibited better salt and drought tolerance [21]. Zhang et al. introduced the *ScABI3* gene from *Syntrichia caninervis* into alfalfa and found that the transgenic alfalfa exhibited better salt tolerance by enhancing antioxidant enzyme activities and improving photosynthetic parameters [22]. Zhang et al. also identified the key gene *P5CDH1* from *C. songorica* and successfully developed transgenic *CsP5CDH1* alfalfa lines. Through drought stress tests, they found that the transgenic lines accumulated large amounts of proline under drought conditions, effectively reducing leaf water loss and enhancing alfalfa’s drought tolerance [23]. Luo et al. developed *MsPPCK1* overexpression plants and demonstrated that the *MsPPCK1* gene could enhance alfalfa’s alkaline tolerance and improve crop yield [24].

Northeast China is one of the key regions for the development of the animal husbandry industry and an important production base for alfalfa [25]. However, alfalfa breeding in the region faces practical challenges, such as the region’s cold climate and saline–alkali soils. A report indicates that when selecting and breeding alfalfa varieties for cultivation in Northeast China, in addition to cold tolerance, it is highly desirable for the varieties to possess other stress-resistance traits, such as drought, salt, and alkali tolerance [26]. Based on this perspective, this study adopted a two-step variety screening process. First, 41 alfalfa varieties were pre-screened for cold tolerance. Then, 15 pre-screened varieties were evaluated for salt tolerance and clustered. Finally, one salt-tolerant alfalfa variety and one salt-sensitive alfalfa variety were selected for further analysis under salt stress. Growth and physiological parameters were measured, and transcriptomic analysis was conducted to reveal the salt-tolerance regulatory mechanisms in alfalfa with contrasting salt tolerance. Furthermore, weighted gene co-expression network analysis (WGCNA) was performed to identify salt-responsive hub genes. This study not only provides a theoretical foundation for the study of alfalfa salt-tolerance mechanisms but also offers valuable insights for molecular breeding of salt-tolerant alfalfa in Northeast China.

## 2. Results

### 2.1. Results of Variety Screening 

#### 2.1.1. Growth and Cold Tolerance Analysis of Different Varieties under Cold Stress

**Figure 1:**
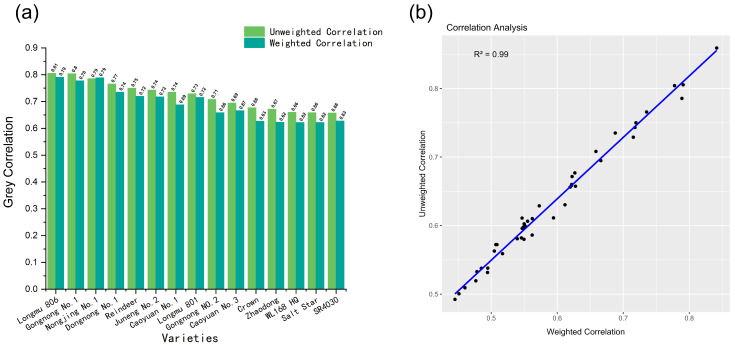
Cold tolerance analysis of alfalfa. (**a**) Grey correlation analysis of cold tolerance in different alfalfa varieties. (**b**) Correlation analysis between weighted correlation and unweighted correlation. Note: For ease of presentation, only the top 15 alfalfa varieties are shown in Figure 1.

In this study, we first performed cold tolerance screening on 41 alfalfa varieties, followed by salt tolerance screening on those with good cold tolerance. As shown in Tables S1–S5, we measured plant height, growth rate, fresh weight, increase in biomass, and dry weight of the 41 alfalfa varieties under cold stress (4 °C) and normal conditions (CK) after 10 days of treatment. To assess cold tolerance, we calculated the relative values of these indicators (Table S6). For a more comprehensive comparison of the cold tolerance of the 41 varieties, we analyzed the relative values of all indicators using grey correlation analysis. As a result, 15 alfalfa varieties were identified (Figure 1a). Correlation analysis between the unweighted correlation and the weighted correlation (Figure 1b) revealed an R^2^ value of 0.99, indicating that both unweighted and weighted correlations can effectively explain the ranking, allowing for further experiments. The grey correlation rankings for all 41 varieties are listed in Table S7.

#### 2.1.2. Analysis of the Half-Lethal Concentration Under Salt Concentrations

**Figure 2:**
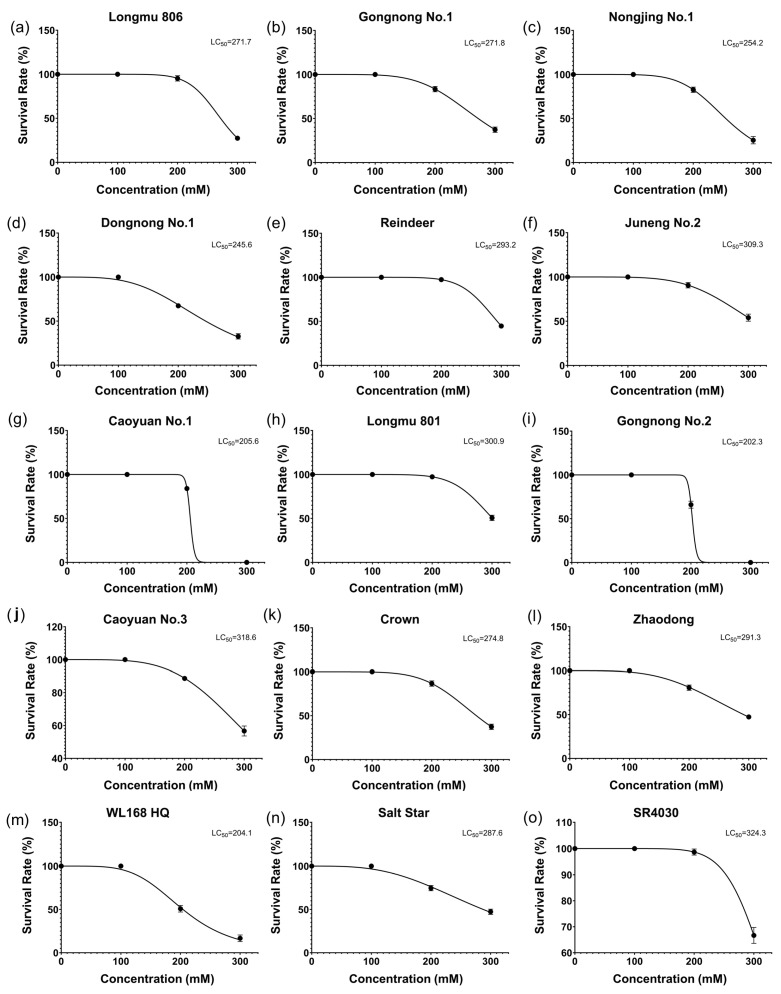
LC_50_ of different alfalfa varieties. (**a**–**o**) represent different alfalfa varieties.

In this study, the mortality rates of 15 alfalfa varieties under different NaCl concentrations (Figure S1) and their respective half-lethal concentrations (LC_50_) (Figure 2) were measured. No mortality was observed at 0 mM and 100 mM NaCl concentrations for any of the varieties, while mortality occurred at both 200 mM and 300 mM NaCl, with a significantly higher rate at 300 mM compared to 200 mM NaCl (Figure S1). Notably, some alfalfa varieties exhibited very low mortality rates at 200 mM NaCl, ranging from 2% to 6%, including Longmu806 (4%), Reindeer (2%), Juneng No.2 (6%), Longmu801 (2%), and SR4030 (2%). The LC_50_ for each variety was calculated based on their mortality rates at 300 mM after 20 days, and the LC_50_ values ranged from 202.3 to 324.3 (Figure 2). The variety with the highest LC_50_ was SR4030, while the lowest was Gongnong No.2.

#### 2.1.3. Cluster Analysis of the Half-Lethal Concentration of Alfalfa

**Figure 3:**
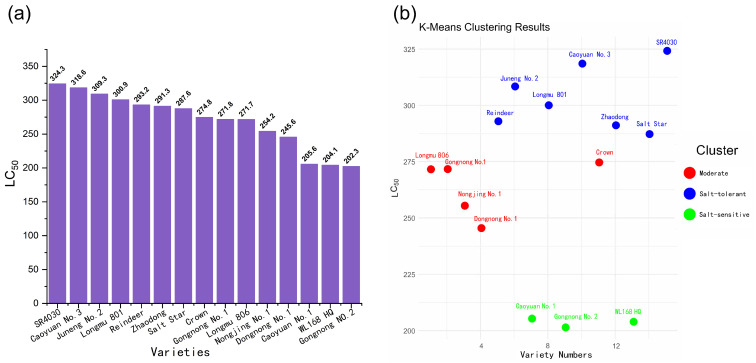
(**a**) LC_50_ ranking of different alfalfa varieties. (**b**) LC_50_ K-means clustering analysis.

Figure 3a presents the LC_50_ values of the tested alfalfa varieties, which are ranked in descending order as follows: SR4030 > Caoyuan No.3 > Juneng No.2 > Longmu801 > Reindeer > Zhaodong > Salt Star > Crown > Gongnong No.1 > Longmu806 > Nongjing No.1 > Dongnong No.1 > Caoyuan No.1 > WL168 HQ (hereinafter referred to as WL168) > Gongnong No.2. K-means clustering analysis was performed based on LC_50_ values (Figure 3b), categorizing the 15 varieties into three groups: salt-tolerant, moderate, and salt-sensitive. Based on this classification, Longmu801 (salt-tolerant) and WL168 (salt-sensitive) were selected for further physiological and transcriptomic analysis.

### 2.2. Effects of Salt Stress on the Growth and Physiology of Alfalfa

#### 2.2.1. Effects of Salt Stress on the Growth Indicators

**Figure 4:**
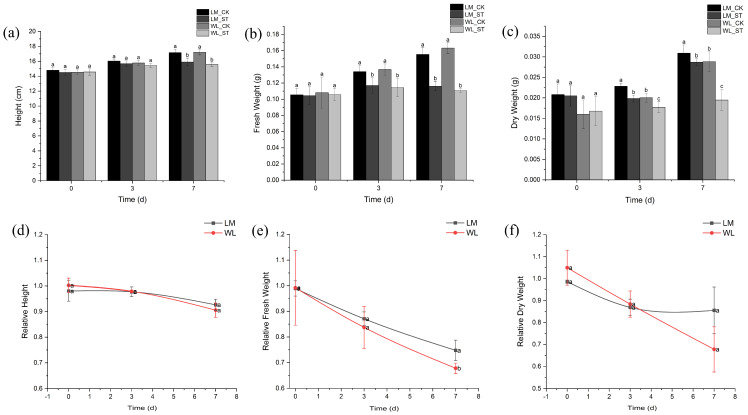
Selected growth indicators of alfalfa: (**a**) height; (**b**) fresh weight; (**c**) dry weight; (**d**) relative height; (**e**) relative fresh weight; and (**f**) relative dry weight. Note: For ease of data presentation and visualization, Longmu801 is abbreviated as LM and WL168 as WL in the Figures. Their respective treatment and control groups are labeled as LM_ST, LM_CK, WL_ST, and WL_CK. In (**a**–**c**), the lowercase letters indicate significant differences among the four different groups at the same time point. In (**d**–**f**), the lowercase letters indicate significant differences between the two varieties at the same time point.

With increasing stress duration, there was no significant difference in plant height between the treatment and control groups of Longmu801 and WL168 on day 3 (Figure 4a). However, by day 7, the plant height in both varieties under stress was significantly lower than that of the control groups, with reductions of 7.38% and 9.50%, respectively, compared to their controls. In Figure 4b, the fresh weight of both varieties decreased significantly compared to their respective controls as stress duration increased. On day 3, the fresh weight of the treatment group for Longmu801 and WL168 decreased by 12.83% and 16.36%, respectively, compared to the control. By day 7, the reductions were 25.30% and 32.24%, respectively. Figure 4c shows that the dry weight of both varieties significantly decreased compared to their controls as the stress duration increased. On day 3, the dry weight of Longmu801 and WL168 decreased by 13.14% and 11.65%, respectively, compared to the control, and by day 7, the reductions were 7.12% and 32.49%, respectively.

To better compare the response differences between Longmu801 and WL168 under salt stress, the relative values of each indicator were calculated as the ratio of the treatment group value to the control group value for the same indicator (i.e., “relative indicator = treatment group (indicator)/control group (indicator)”), to minimize the differences caused by the variety baseline. Figure 4d illustrates that relative plant height showed a decreasing trend as stress duration increased, but there was no significant difference between the two varieties at different time points. In Figure 4e, relative fresh weight also decreased with increasing stress duration. While no significant differences were observed on day 3, by day 7, Longmu801 had a significantly higher relative fresh weight than WL168, being 110.28% of WL168’s relative fresh weight. Relative dry weight decreased with increasing stress duration but there were no significant differences between the two varieties at any time point (Figure 4f).

#### 2.2.2. Effects of Salt Stress on the Photosynthetic Pigments

**Figure 5:**
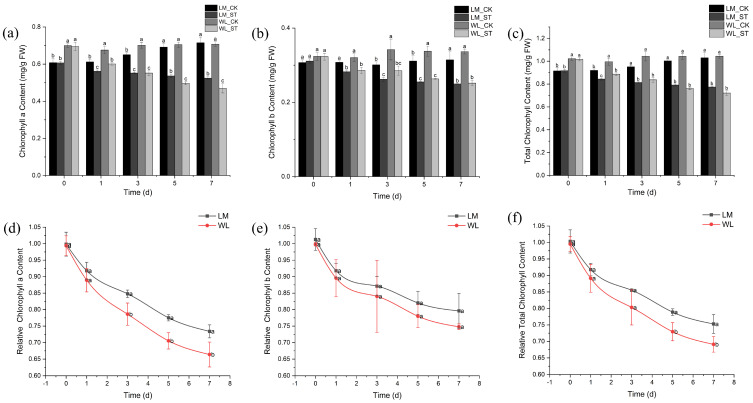
Selected photosynthetic pigment content of alfalfa: (**a**) chlorophyll a content; (**b**) chlorophyll b content; (**c**) total chlorophyll content; (**d**) relative chlorophyll a content; (**e**) relative chlorophyll b content; and (**f**) relative total chlorophyll content. In (**a**–**c**), the lowercase letters indicate significant differences among the four different groups at the same time point. In (**d**–**f**), the lowercase letters indicate significant differences between the two varieties at the same time point.

The chlorophyll a content of Longmu801 and WL168 decreased as the duration of stress increased, with the treatment group showing significantly lower levels than the control group at all time points (Figure 5a). The most significant differences were observed on day 7, with reductions of 26.64% and 33.63%, respectively, compared to the control. Figure 5b illustrates a similar decreasing trend in chlorophyll b content, where the treatment groups were significantly lower than the controls at all time points. On day 7, the most significant reductions of 20.62% and 25.19% were observed. The total chlorophyll content also exhibited a decreasing trend over time, with significant reductions at each time point (Figure 5c). On day 7, the treatment groups were reduced by 24.80% and 30.91%, respectively, compared to the control. The relative chlorophyll a content of Longmu801 and WL168 decreased with the duration of stress (Figure 5d). From day 3 onward, a significant difference was observed between the two varieties, with Longmu801 showing 7.85%, 9.92%, and 10.58% higher relative chlorophyll a content than WL168 on days 3, 5, and 7, respectively. The relative chlorophyll b content of both varieties also decreased with the duration of stress (Figure 5e), but no significant differences between the varieties were observed at any time point. The relative total chlorophyll content of both varieties decreased over time, with significant differences appearing on days 5 and 7 (Figure 5f). On these days, Longmu801 had 8.13% and 8.95% higher relative total chlorophyll content than WL168, respectively.

#### 2.2.3. Effects of Salt Stress on Oxidative Stress Markers

**Figure 6:**
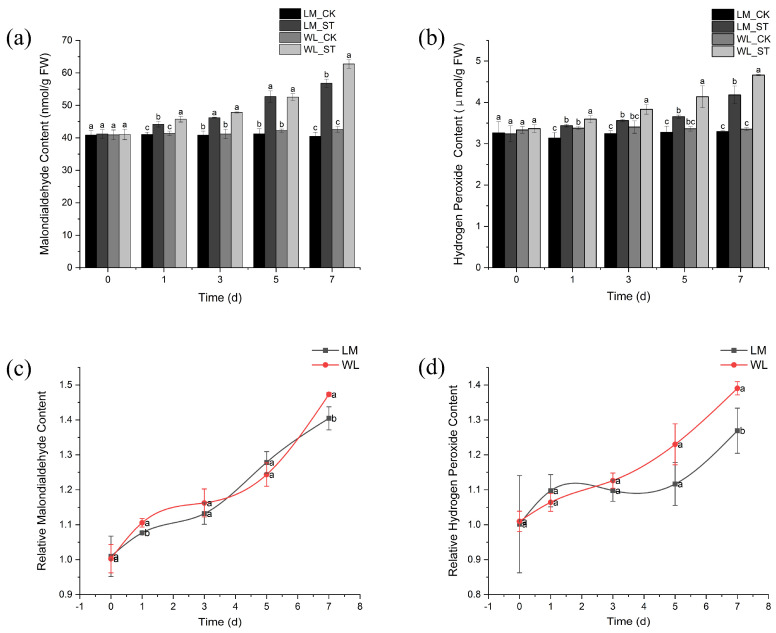
Selected oxidative stress marker content of alfalfa: (**a**) MDA content; (**b**) H_2_O_2_ content; (**c**) relative MDA content; and (**d**) relative hydrogen peroxide content. In (**a**,**b**), the lowercase letters indicate significant differences among the four different groups at the same time point. In (**c**,**d**), the lowercase letters indicate significant differences between the two varieties at the same time point.

The malondialdehyde (MDA) content of Longmu801 and WL168 increased as the duration of stress increased, with the treatment groups significantly higher than their respective controls at all time points (Figure 6a). The most significant difference was observed on day 7, with the MDA content of Longmu801 and WL168 increasing by 40.4% and 47.29%, respectively, compared to the controls. On days 1 and 7, WL168 had significantly higher MDA content than Longmu801. As shown in Figure 6b, the hydrogen peroxide (H_2_O_2_) content of both Longmu801 and WL168 also increased over time, with the treatment groups significantly higher than the controls at all time points. The most pronounced difference was on day 7, with the H_2_O_2_ content in the salt-treated Longmu801 and WL168 groups increasing by 26.92% and 39.03%, respectively, compared to their controls. Additionally, on days 1, 3, 5, and 7, WL168 had significantly higher H_2_O_2_ content than Longmu801. The relative MDA content of both varieties increased over time, with WL168 showing significantly higher values than Longmu801 on days 1 and 7, by 2.68% and 4.87%, respectively (Figure 6c). The relative H_2_O_2_ content of both varieties also increased over time, with WL168 showing a significantly higher relative H_2_O_2_ content than Longmu801 on day 7, with an increase of 9.55% (Figure 6d).

#### 2.2.4. Effects of Salt Stress on Osmolytes

**Figure 7:**
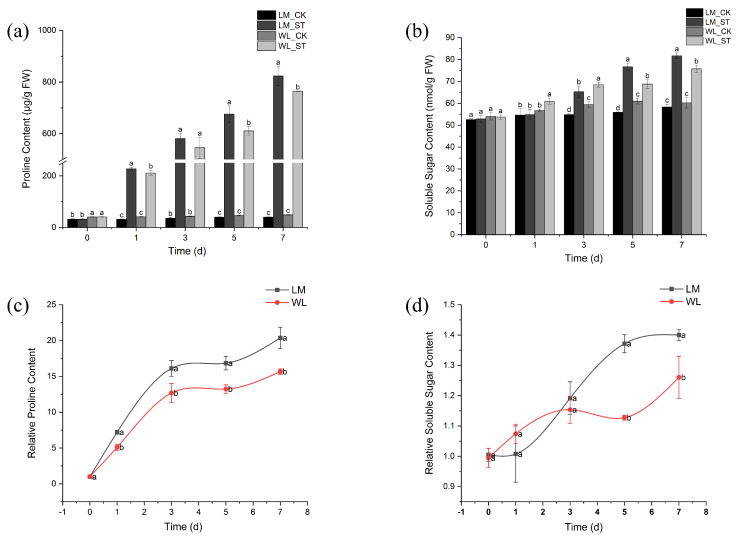
Selected osmolyte content of alfalfa: (**a**) proline content; (**b**) soluble sugar content; (**c**) relative proline content; and (**d**) relative soluble sugar content. In (**a**,**b**), the lowercase letters indicate significant differences among the four different groups at the same time point. In (**c**,**d**), the lowercase letters indicate significant differences between the two varieties at the same time.

The proline content of Longmu801 and WL168 increased with the duration of salt stress (Figure 7a). The proline content in the treatment groups was significantly higher than that in the control groups at all time points, with the most significant difference observed on day 7, where proline levels in the treatment groups of Longmu801 and WL168 were 19.35 and 14.67 times higher than their respective controls. On days 5 and 7, the proline content in Longmu801 was significantly higher than in WL168. The soluble sugar content also increased over time (Figure 7b). On day 1, the soluble sugar content in the WL168 salt treatment group was significantly higher than in the control group, while no significant change was observed in Longmu801. On days 3, 5, and 7, the soluble sugar content in the treatment groups of both varieties was significantly higher than in their respective controls, with the most significant difference on day 7, where the salt treatment groups of Longmu801 and WL168 showed increases of 40% and 25.87%, respectively, compared to their controls. The relative proline content of both varieties increased with the duration of stress (Figure 7c). On day 1, the relative proline content of Longmu801 was significantly higher than that of WL168, reaching a maximum on day 7, with an increase of 29.96% compared to WL168. The relative soluble sugar content also increased over time (Figure 7d). On days 5 and 7, the relative soluble sugar content of Longmu801 was significantly higher than that of WL168, with the most significant difference observed on day 5, showing an increase of 21.67%.

#### 2.2.5. Effects of Salt Stress on Antioxidant Enzymes

**Figure 8:**
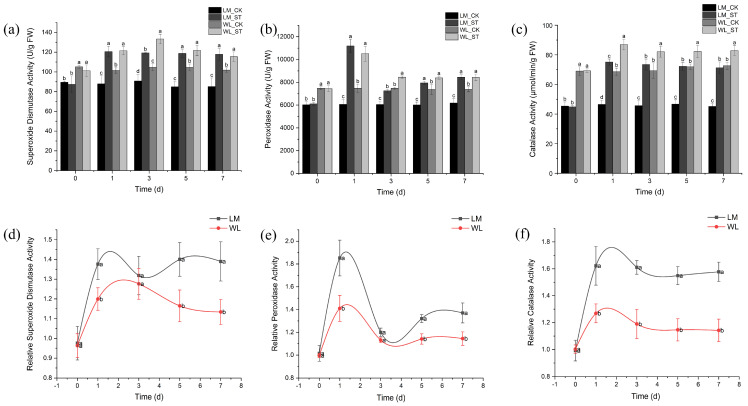
Selected antioxidant enzymes activity of alfalfa: (**a**) SOD activity; (**b**) POD activity; (**c**) CAT activity; (**d**) relative SOD activity; (**e**) relative POD activity; and (**f**) relative CAT activity. In (**a**–**c**), the lowercase letters indicate significant differences among the four different groups at the same time point. In (**d**–**f**), the lowercase letters indicate significant differences between the two varieties at the same time point.

The superoxide dismutase (SOD) activity of Longmu801 and WL168 remained at a relatively high level as stress duration increased (Figure 8a). On day 1, the SOD activity of the treatment groups was higher than their respective controls, with Longmu801 increasing by 37.19% compared to the control. WL168 reached its peak SOD activity on day 3, increasing by 27.49% compared to the control, but showed a decreasing trend on days 5 and 7. Longmu801, on the other hand, maintained a consistent SOD activity throughout the time points, remaining significantly higher than the control group. The peroxidase (POD) activity of Longmu801 and WL168 initially increased and then decreased over time (Figure 8b). On day 1, the POD activity of the treatment groups was highest, with Longmu801 and WL168 increasing by 84.63% and 40.65%, respectively, compared to their controls. Both varieties showed a decreasing trend on days 3, 5, and 7, but their POD activity remained significantly higher than their respective controls. Figure 8c shows that catalase (CAT) activity remained at a high level as stress duration increased, with both Longmu801 and WL168 reaching their highest CAT activity on day 1, increasing by 61.68% and 26.8%, respectively, compared to the control. A decreasing trend followed, but CAT activity remained significantly higher than the control in both varieties. The relative SOD activity of both Longmu801 and WL168 was significantly higher than on day 0 (Figure 8d). On days 1, 5, and 7, Longmu801 had significantly higher relative SOD activity than WL168, while no significant difference was observed on day 3. Figure 8e shows a similar trend in relative POD activity, with Longmu801 showing a 31.36% higher relative POD activity than WL168 on day 1. No significant difference was observed on day 3, but Longmu801 had significantly higher POD activity on days 5 and 7. The relative CAT activity of both varieties was higher than on day 0, with Longmu801 showing significantly higher CAT activity than WL168 at all time points (Figure 8f).

#### 2.2.6. Comprehensive Grey Correlation Analysis Based on Physiological Indicators

**Figure 9:**
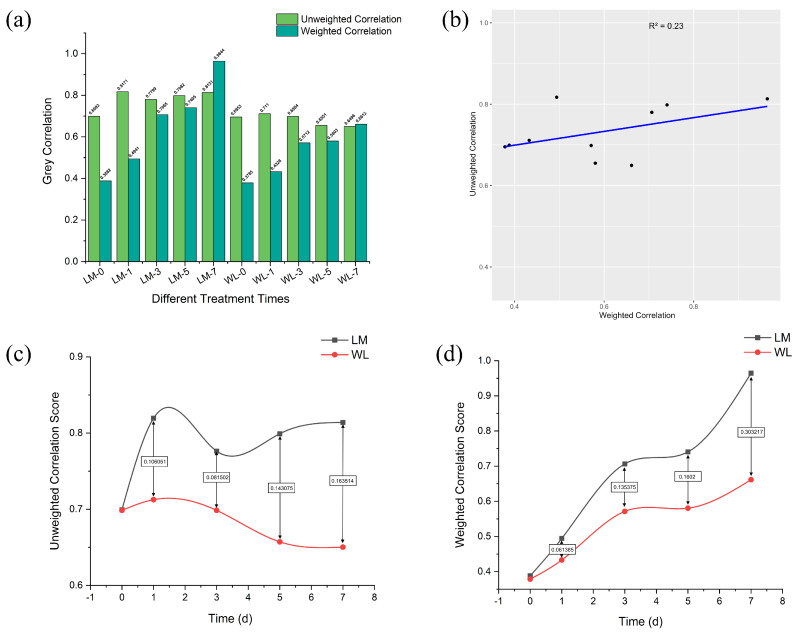
(**a**) Grey correlation of different time points. (**b**) Correlation between unweighted and weighted correlation degrees. (**c**) Unweighted correlation at different time points. (**d**) Weighted correlation at different time points. Note: In (**a**), LM-0,1,3,5,7 and WL-0,1,3,5,7 represent the different time points for the two varieties.

To determine the key time point for transcriptomic sequencing, this study conducted a grey correlation analysis of physiological indicators. First, the unweighted correlation ranking was obtained for each time point. The critic method was then used to calculate weighted values, and based on this, the weighted correlation was determined. The ranking for each time point is shown in Figure 9a. Next, Pearson correlation analysis was performed between the equal-weight and weighted grey correlation degrees, with the results showing a low correlation (R^2^ = 0.23) in Figure 9b, indicating that the weight values had a significant impact on the grey correlation. Therefore, data from both methods were considered in decision-making. Figure 9c,d show the differences between the unweighted and weighted correlation at different time points. The largest difference was observed on day 7 (0.163514 and 0.303217), while the smallest difference was on day 0. After comprehensive consideration, day 7 was chosen as the sampling time point for transcriptomic sequencing.

### 2.3. Transcriptomic Analysis of Different Alfalfa Varieties Under Salt Stress

#### 2.3.1. Overview of Transcriptomic Sequencing Statistics

First, cDNA libraries were constructed for the experimental and control groups of different varieties under salt stress, and transcriptomic sequencing was performed using the Illumina Hiseq 4000 platform, with RNA-seq used to obtain transcriptomic data (details of the specific methods can be found in Section 4.4). The total number of raw reads ranged from 35,512,472 to 43,229,200, and the sequencing data size ranged from 5.33 G to 6.48 G (Table S8). After filtering, the number of valid reads ranged from 33,821,714 to 41,592,130, with a sequencing data size between 5.07 G and 6.24 G. The valid ratio was between 94.24% and 96.91%, Q20 and Q30 values were 99.20% to 99.44% and 96.59% to 97.50%, respectively, and the GC content of the samples ranged from 41.5% to 42.50%, with an average GC content of 42%.

The valid data were aligned to the reference genome using Hisat (details of the specific methods can be found in Section 4.4) after preprocessing. First, all available alfalfa reference genomes were used for alignment. The average alignment rate with the “Zhongmu No. 4” genome was slightly higher than that of the “Xinjiang Daye” genome at 87.17% and significantly higher than the “Zhongmu No. 1” genome at 71.32% (Table S9) [27]. Therefore, the “Zhongmu No. 4” alfalfa reference genome was selected for further analysis. As shown in Table S10, by aligning the valid reads to the reference genome, the genomic alignment for each sample was obtained, allowing the identification of valid reads’ positions on the reference genome as well as specific sequence information. The aligned reads (mapped reads) ranged from 30,258,802 to 34,413,892, with an alignment rate between 84.21% and 89.94% and an average alignment rate of 87.38%. An alignment rate greater than 80% indicates that the selected reference genome is appropriate and the alignment was effective. Of these, the proportion of uniquely mapped reads (unique mapped) ranged from 36.98% to 39.54%, the proportion of paired-end mapped reads (PE mapped) ranged from 72.21% to 78.28%, non-spliced reads ranged from 22.46% to 25.04%, and spliced reads ranged from 13.81% to 15%.

**Figure 10:**
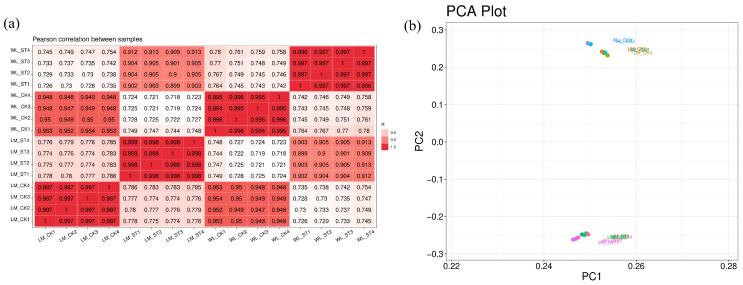
(**a**) Correlation analysis between samples and (**b**) principal component analysis (PCA) between samples.

For ease of data processing, Longmu801 is abbreviated as LM, and WL168 is abbreviated as WL in the transcriptomic analysis. Their respective treatment and control groups are LM_ST, LM_CK, WL_ST, and WL_CK. The gene expression levels of the samples in the different treatment groups are represented by darker colors, with correlation coefficients close to 1, indicating a high correlation among the samples within each group (Figure 10a). From Figure 10b, it can be observed that in the Principal Component Analysis (PCA), there is a larger degree of dispersion between different varieties, such as LM_CK and WL_CK, compared to LM_ST and WL_ST, which is due to the different experimental treatment conditions. The relatively smaller dispersion between LM_CK and WL_CK, as well as between LM_ST and WL_ST.

#### 2.3.2. Transcriptomic Analysis of Longmu801 and WL168 Under Salt Stress

**Figure 11:**
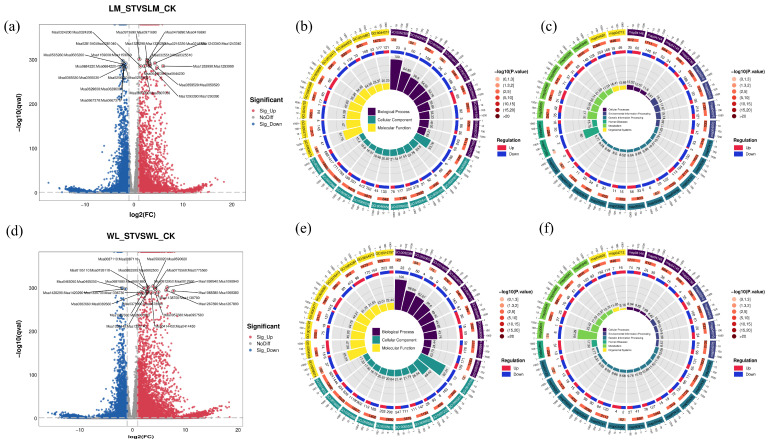
Transcriptomic Analysis of Longmu801 and WL168 under Salt Stress. (**a**) Differentially Expressed Genes in Longmu 801. (**b**) GO Enrichment Function of Longmu801. (**c**) KEGG Pathways of Longmu801. (**d**) Differentially Expressed Genes in WL168. (**e**) GO Enrichment Function of WL168. (**f**) KEGG Pathways of WL168.

There were 16,485 differentially expressed genes (DEGs) in the LM_ST vs. LM_CK group, of which 5929 DEGs were upregulated and 10,556 DEGs were downregulated (Figure 11a). In the group, a total of 14,350 DEGs were annotated with GO functions, which were enriched in 3568 GO functions. These functions are classified into three categories: 1917 in Biological Process, 438 in Cellular Component, and 1213 in Molecular Function. A total of 799 GO functions were significantly enriched (*p* < 0.05). The GO functions based on *p*-values, including kinesin complex (GO:0005871), microtubule-based movement (GO:0007018), plant-type secondary cell wall biogenesis (GO:0009834), extracellular region (GO:0005576), microtubule motor activity (GO:0003777), and cell wall (GO:0005618), among others (Figure 11b). A total of 5396 DEGs were annotated into 135 KEGG pathways. These 135 pathways are classified into six primary metabolic categories: 4 in Environmental Information Processing, 4 in Cellular Processes, 21 in Genetic Information Processing, 2 in Human Diseases, 102 in Metabolism, and 2 in Organismal Systems. A total of 37 KEGG pathways were significantly enriched (*p* < 0.05). The pathways based on *p*-values are displayed, including the KOK signaling pathway—plant (ko04016), ether lipid metabolism (ko00565), glutathione metabolism (ko00480), sphingolipid metabolism (ko00600), plant–pathogen interaction (ko04626), and phenylpropanoid biosynthesis (ko00940), among others (Figure 11c).

Figure 11d illustrates that the WL_ST vs. WL_CK group contained a total of 18,726 DEGs, with 7798 showing upregulation and 10,928 exhibiting downregulation. In the group, a total of 16,307 DEGs were annotated with GO functions, enriching 3817 GO entries. These entries are classified into three categories: 2052 in Biological Process, 473 in Cellular Component, and 1292 in Molecular Function. A total of 782 GO functions were significantly enriched (*p* < 0.05). The GO functions based on *p*-values, including plasma membrane (GO:0005886), kinesin complex (GO:0005871), microtubule-based movement (GO:0007018), plant-type secondary cell wall biogenesis (GO:0009834), extracellular region (GO:0005576), and microtubule motor activity (GO:0003777), among others (Figure 11e). A total of 6043 DEGs were annotated into 133 KEGG pathways. These 133 pathways are classified into six primary metabolic categories: 4 in Environmental Information Processing, 4 in Cellular Processes, 21 in Genetic Information Processing, 2 in Human Diseases, 100 in Metabolism, and 2 in Organismal Systems. A total of 39 KEGG pathways were significantly enriched (*p* < 0.05). KEGG pathways based on *p*-values are displayed, including ether lipid metabolism (ko00565), glutathione metabolism (ko00480), ABC transporters (ko02010), arginine and proline metabolism (ko00330), glycerolipid metabolism (ko00561), and isoflavonoid biosynthesis (ko00943), among others (Figure 11e).

#### 2.3.3. Analysis of DEGs Between Longmu801 and WL168

**Figure 12:**
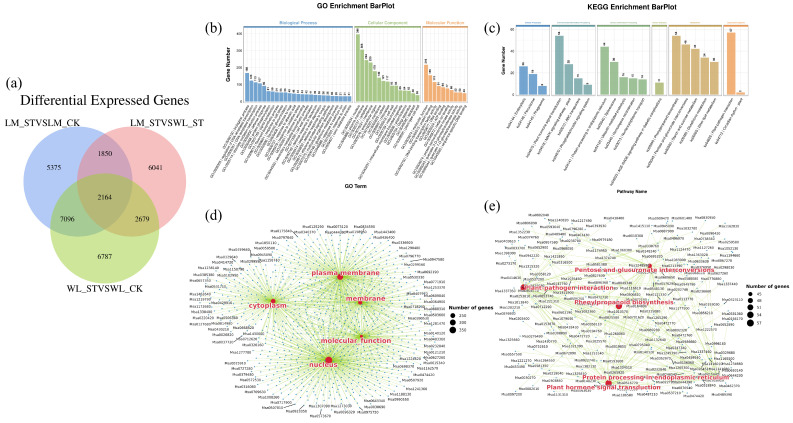
Analysis of DEGs between Longmu801 and WL168. (**a**) DEGs Venn diagrams. (**b**) GO functions of 2164 DEGs. (**c**) KEGG pathways of 2164 DEGs. (**d**) The relationship between GO functions and DEGs. (**e**) The relationship between KEGG pathways and DEGs.

To better explore the differences in response to salt stress between the varieties, Venn diagrams were generated for the different comparison groups, as shown in Figure 12a. Among the groups LM_ST vs. LM_CK, LM_ST vs. WL_ST, and WL_ST vs. WL_CK, a total of 2164 DEGs were common across all comparisons. These 2164 DEGs represent genes that respond to salt stress in both varieties. There are 1850 DEGs that are shared between LM_ST vs. LM_CK and LM_ST vs. WL_ST but are absent in WL_ST vs. WL_CK, indicating that these 1850 DEGs are specifically responsive to salt stress in the LM variety but not in the WL variety. The expression of these genes in LM is significantly different from that in WL, suggesting that these 1850 DEGs are unique to the LM variety’s response to salt stress. Additionally, there are 2679 DEGs that are shared between WL_ST vs. WL_CK and LM_ST vs. WL_ST but are absent in LM_ST vs. LM_CK. These 2679 DEGs are specifically responsive to salt stress in the WL variety but not in the LM variety, and their expression levels in WL are significantly different from those in LM, indicating that these 2679 DEGs are unique to the WL variety’s response to salt stress.

As shown in Figure 12b, the 2164 shared DEGs were enriched in 1291 GO functions, which can be categorized into three groups: 689 in Biological Process, 150 in Cellular Component, and 452 in Molecular Function. Among these, 27 GO functions were significantly enriched (*p* < 0.05). The GO functions under the Biological Process category include: biological process (GO:0008150), regulation of DNA-templated transcription (GO:0006355), DNA-templated transcription (GO:0006351), obsolete oxidation-reduction process (GO:0055114), defense response (GO:0006952), and others. Under the Cellular Component category, the GO functions include: nucleus (GO:0005634), plasma membrane (GO:0005886), cytoplasm (GO:0005737), membrane (GO:0016021), and extracellular region (GO:0005576), among others. Under the Molecular Function category, the GO functions include: molecular function (GO:0003674), protein binding (GO:0005515), DNA-binding transcription factor activity (GO:0003700), DNA binding (GO:0003677), and ATP binding (GO:0005524), among others. For the relationship between the GO functions and DEGs, the top five GO functions with a *p*-value < 0.05 were selected for display (Figure 12d).

As shown in Figure 11c, these DEGs were enriched in 111 KEGG pathways, of which 85 were significantly enriched (*p* < 0.05). These include plant–pathogen interaction (ko04626); plant hormone signal transduction (ko04075); phenylpropanoid biosynthesis (ko00940); pentose and glucuronate interconversions (ko00040), folding, sorting, and degradation in the endoplasmic reticulum (ko04141); starch and sucrose metabolism (ko00500); glutathione metabolism (ko00480); spliceosome (ko03040); ether lipid metabolism (ko00565); and glycerolipid metabolism (ko00561), among others. The relationship between the KEGG pathways and DEGs, the top five GO functions with a *p*-value < 0.05 were selected for display (Figure 12e). The GO function enrichment and KEGG pathway enrichment for the 1850 DEGs and 2679 DEGs are shown in the Supplementary Figures (Figures S2 and S3).

#### 2.3.4. Results of qRT-PCR Validation

In the transcriptomic analysis, we identified a class of genes referred to as “abscisic acid and environmental stress-inducible proteins”, which exhibited significant changes following salt treatment. These genes, *Msa0819320*, *Msa0738880*, *Msa0783290*, *Msa0819370*, and *Msa0783280*, were selected as candidate genes for further validation using qPCR technology (Figure S4a). A correlation analysis between the transcriptomic (RNA-seq) results and qPCR was conducted, with an R^2^ value of 0.99, indicating that the transcriptomic results are reliable (Figure S4b).

### 2.4. Weighted Gene Co-Expression Network Analysis

#### 2.4.1. Sample Processing and WGCNA Module Correlation Analysis

**Figure 13:**
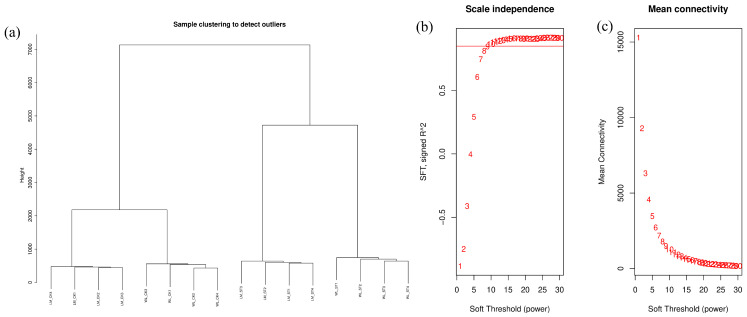
(**a**) Clustering analysis of different samples. (**b**,**c**) Soft threshold and gene connectivity.

To discover additional core genes involved in the response to salt stress, we conducted a weighted gene co-expression network analysis (WGCNA). First, genes from the transcriptomic analysis with an expression level below 2 were filtered out, resulting in 32,684 genes being included in the WGCNA. A sample clustering dendrogram was then constructed from the expression levels of these genes across 16 samples to assess for any potential outliers. All samples from the different groups clustered well, indicating no outliers, and thus, no samples needed to be removed (Figure 13a). In this WGCNA analysis, the plateau threshold was set to 0.85, and the soft threshold was set to 1. In Figure 13b, the *x*-axis represents the soft threshold, and the *y*-axis represents the evaluation parameter of the scale-free network, which indicates the squared correlation coefficient of the corresponding network. In Figure 13c, the *x*-axis represents the soft threshold, and the *y*-axis represents the average connectivity (adjacency function) of all genes under different β values.

**Figure 14:**
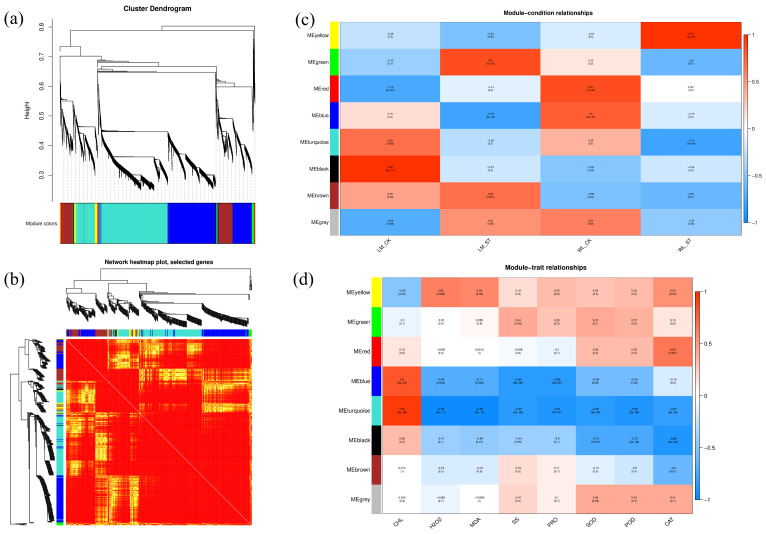
Module correlation analysis. (**a**) Module hierarchical clustering dendrogram. (**b**) Module correlation. (**c**) Module–sample correlation. (**d**) Module–physiological correlation.

To increase biological relevance, the topological overlap measure (TOM) was used to calculate the degree of association between genes. In addition to analyzing the relationship between two genes, the connections between these two genes and other genes were also considered. The minimum module size for module detection was set to 30. In this WGCNA analysis, genes were clustered into eight modules: green, yellow, red, blue, turquoise, black, brown, and grey (Figure 14a). The grey module contains all unclustered genes and thus holds little analytical significance, so it was not analyzed in this study. Figure 14b shows the correlation analysis between the genes within each module. Generally, genes within the same module are represented by deeper colors, while the color between modules is lighter. The consistent color representation of genes within each module indicates good clustering, offering greater research value and potential insights.

A correlation analysis was conducted between different modules, experimental groups, and physiological indicators. Figure 14c illustrates the correlation between different modules and experimental groups. The color variations across modules and groups reflect the strength of these correlations, enabling a visual assessment of gene associations within each module. The values in parentheses denote *p*-values, with smaller values indicating higher statistical significance. Figure 14d displays the correlation between different modules and physiological indicators. These correlations divide the modules into two distinct groups: one group, including the yellow, green, and red modules, shows positive correlations with indicators such as soluble sugars (SS), proline (PRO), superoxide dismutase (SOD), peroxidase (POD), and catalase (CAT). The other group, comprising the blue, turquoise, and black modules, is negatively correlated with hydrogen peroxide (H2O2) and malondialdehyde (MDA). Further analysis will focus on these six modules.

#### 2.4.2. Study of Modules Gene Functions and Hub Gene Screening

**Figure 15:**
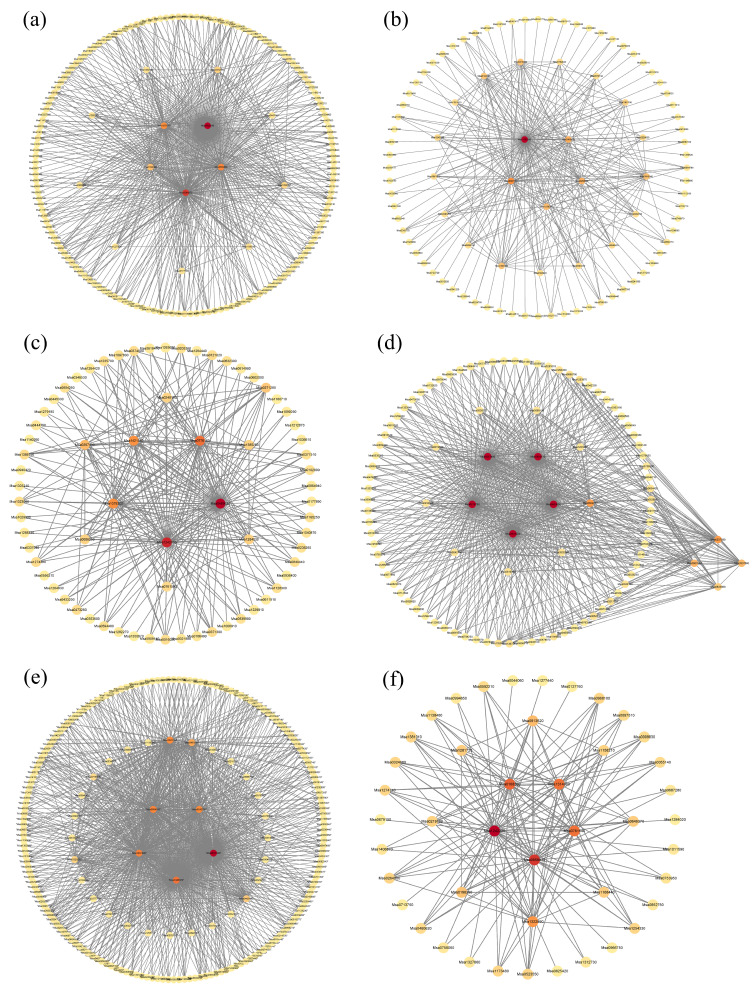
Hub genes of different modules. (**a**) Hub genes in yellow modules. (**b**) Hub genes in green modules. (**c**) Hub genes in red modules. (**d**) Hub genes in blue modules. (**e**) Hub genes in turquoise modules. (**f**) Hub genes in black modules. Note: The hub genes were clustered using the Mcode plugin in Cytoscape (Details of the specific methods can be found in Section 4.6.), and the top five genes with the highest degree values within each cluster were selected. The darker the color, the higher the degree value.

Figure S5 provides an overview of the GO functions and KEGG pathways enriched in genes from six distinct color modules. The yellow module is significantly enriched for 331 GO functions, including cytoplasm (GO:0005737), chloroplast (GO:0009507), cytosol (GO:0005829), response to cold (GO:0009409), and protein transport (GO:0015031). The yellow module is also significantly enriched for 51 KEGG pathways, including starch and sucrose metabolism (ko00500), protein export (ko03060), phenylpropanoid biosynthesis (ko00940), and protein processing in the endoplasmic reticulum (ko04141). The hub genes identified in this module are: *Msa0835190*, *Msa0644060*, *Msa0559800*, *Msa1079730*, and *Msa0192380* (Figure 15a).

The green module is significantly enriched for 291 GO functions, including cytosol (GO:0005829), metal ion binding (GO:0046872), chloroplast thylakoid membrane (GO:0009535), plastoglobuli (GO:0010287), P-body (GO:0000932), and photosynthesis (GO:0009768). The green module is also significantly enriched for 49 KEGG pathways, including propanoate metabolism (ko00640), photosynthesis—antenna proteins (ko00196), glycolysis/gluconeogenesis (ko00010), and valine, leucine, and isoleucine degradation (ko00280). Figure 15b presents the network diagram of hub genes in the green module, with the selected hub genes being: *Msa1168040*, *Msa0367140*, *Msa0458690*, *Msa0066410*, and *Msa125874*.

The red module is significantly enriched for 215 GO functions, including cytosol (GO:0005829), cytosolic large ribosomal subunit (GO:0022625), nucleus (GO:0005634), mitochondrion (GO:0005739), and plastid (GO:0009536). The red module is also significantly enriched for 38 KEGG pathways, including glycolysis/gluconeogenesis (ko00010), phenylpropanoid biosynthesis (ko00940), fatty acid elongation (ko00062), oxidative phosphorylation (ko00190), and tryptophan metabolism (ko00380). Figure 15c presents the network diagram of hub genes in the red module, with the selected hub genes being: *Msa0489620*, *Msa1242210*, *Msa0776160*, *Msa1078300*, and *Msa0397400*.

The GO enrichment analysis for genes in the blue module revealed 1171 significantly enriched GO functions, including catalase activity (GO:0004096), xanthophyll binding (GO:0051738), glyceraldehyde-3-phosphate dehydrogenase (NADP+) (phosphorylating) activity (GO:0047100), peroxiredoxin activity (GO:0051920), and antioxidant activity (GO:0016209). The KEGG enrichment analysis of the blue module identified 115 significantly enriched pathways, including plant hormone signal transduction (ko04075), ribosome (ko03010), protein processing in the endoplasmic reticulum (ko04141), nucleocytoplasmic transport (ko03013), and endocytosis (ko04144). As shown in Figure 15d, genes in the blue module were clustered into two groups, with the second cluster having the highest degree values. The top five genes based on degree ranking were selected as hub genes: *Msa0933220*, *Msa0850110*, *Msa0605650*, *Msa0257490*, and *Msa0218780*.

The GO enrichment analysis for genes in the turquoise module revealed 1306 significantly enriched functions, including nucleus (GO:0005634), cytoplasm (GO:0005737), plasma membrane (GO:0005886), protein binding (GO:0005515), membrane (GO:0016021), and biological process (GO:0008150). The KEGG enrichment analysis of the turquoise module identified 113 significantly enriched pathways, including plant hormone signal transduction (ko04075), ribosome (ko03010), amino sugar and nucleotide sugar metabolism (ko00520), phagosome (ko04145), starch and sucrose metabolism (ko00500), and MAPK signaling pathway—plant (ko04016). Figure 15e presents the network diagram of hub genes in the turquoise module, with the top five genes based on degree ranking selected as hub genes: *Msa0631990*, *Msa1335970*, *Msa1031990*, *Msa0564100*, and *Msa0242790*.

The GO enrichment analysis for genes in the black module revealed 192 significantly enriched functions, including inorganic triphosphate phosphatase activity (GO:0050355), structural constituent of ribosome (GO:0003735), translation (GO:0006412), and cellular response to singlet oxygen (GO:0071452). The KEGG enrichment analysis of the black module identified 35 significantly enriched pathways, including ribosome (ko03010), spliceosome (ko03040), purine metabolism (ko00230), pyrimidine metabolism (ko00240), photosynthesis (ko00195), and flavonoid biosynthesis (ko00941). Figure 15f presents the network diagram of hub genes in the black module, with the top five genes based on degree ranking selected as hub genes: *Msa1242220*, *Msa0958830*, *Msa0168360*, *Msa1314080*, and *Msa0761640*.

#### 2.4.3. Correlation Analysis of HUB Genes

**Figure 16:**
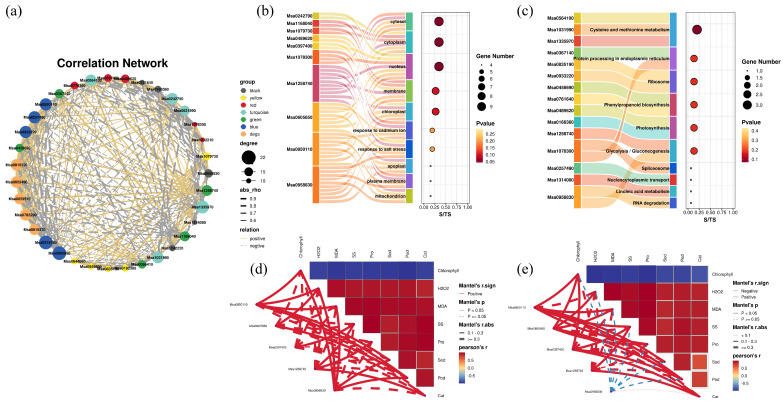
Analysis of HUB Genes. (**a**) Co-expression network relationship of hub genes. (**b**) GO enrichment Sankey diagram of hub genes. (**c**) KEGG enrichment Sankey diagram of hub genes. (**d**) Relationship between hub genes responding to salt stress and physiological indicators in Longmu801. (**e**) Relationship between hub genes responding to salt stress and physiological indicators in WL168.

The correlation analysis of the 35 hub genes identified from the six different WGCNA color modules and those annotated as “abscisic acid and environmental stress-inducible proteins” in the transcriptomic analysis revealed that the genes *Msa0218780* and *Msa0605650* have the highest degree values, with 22 hub genes co-expressed with these two genes. In contrast, *Msa1242210*, *Msa1078300*, and *Msa1314080* have the lowest degree values, with only 10 hub genes co-expressed (Figure 16a). The blue module exhibited the highest co-expression connectivity, while the yellow and black modules had lower connectivity. Figure 16b,c show the GO and KEGG enrichment analysis of the selected hub genes. The GO term “response to salt stress” (GO:0009651) was enriched, including five hub genes: *Msa085011*, *Msa0605650*, *Msa0397400*, *Msa1258740*, and *Msa0958830*.

A Mantel test was conducted to analyze the correlation between these five hub genes and physiological indicators (Figure 16d,e). In Longmu801, H_2_O_2_, MDA, SS, Pro, Sod, Pod, and Cat were negatively correlated with chlorophyll content. All five hub genes showed a positive correlation with the physiological indicators. In WL168, the same physiological indicators were negatively correlated with chlorophyll content. Specifically, *Msa0958830* was negatively correlated with H_2_O_2_, MDA, SS, Pro, Sod, and Pod but positively correlated with Cat, although this correlation was weak. The remaining hub genes showed positive correlations with the physiological indicators.

## 3. Discussion

### 3.1. Interpretation of Variety Screening Outcomes

In this study, we adopted the research approach proposed by Li et al. [26], which suggests that, based on a certain level of resistance, further screening for other resistance genes can help identify alfalfa resistance genes better suited to the local ecological environment. Therefore, we implemented a two-stage screening process: the first stage involved cold tolerance screening, and the second focused on salt tolerance.

In the first stage, a series of growth indicators, such as growth rates and fresh weight, were measured. Although these are standard growth indicators, they effectively reflect the growth and development of plants under 4 °C stress [28,29]. To rank the cold tolerance of different varieties, we employed a grey correlation analysis, a method widely recognized for its effectiveness in integrating multiple indicators for comprehensive ranking. This approach has already been extensively applied in plants, such as tea (*Camellia sinensis* L.) [30], soybean (*Glycine max* L.) [31], and potato (*Solanum tuberosum* L.) [32]. Two ranking modes were generated: weighted grey correlation degree [33] and unweighted grey correlation degree, and 15 varieties were selected for further salt tolerance screening. We applied a subjective–objective approach to screen the 15 alfalfa varieties. Subjectively, we selected varieties that were already cultivated in Northeast China, such as Longmu801, Longmu806, Gongnong No.1, Gongnong No.2, and Zhao Dong [34,35]. Objectively, we used grey correlation degree scores for the screening process. By combining subjective and objective selection, we concluded that these 15 varieties possess cold tolerance.

In the second stage, we evaluated the salt tolerance of different 15 alfalfa varieties using the half-lethal rate [36] under salt stress to ensure the rationality and purposefulness of the experimental data. Assessing plant resistance using the half-lethal concentration is a well-established method. For example, Yang et al. evaluated cold tolerance in various Liliaceae species by measuring cell mortality [37]. We treated 15 alfalfa varieties with different salt concentrations (0, 100, 200, and 300 mM) and observed that, as the duration of stress increased, a large number of plants died under 300 mM salt stress. After prolonged treatment, some deaths also occurred under 200 mM, but the mortality rate was significantly lower. No deaths were observed under 100 mM and 0 mM conditions. The absence of mortality at 0 mM was expected and indicates that external environmental factors did not affect the experimental results. Similarly, the lack of mortality under 100 mM suggests that alfalfa, being a highly adaptable species with inherent salt tolerance [38], gradually adjusted to the 100 mM environment over time, leading to no deaths. Mortality occurred at both 200 mM and 300 mM, indicating that, over time, the stress exerted by these concentrations exceeded the plant’s tolerance limits, resulting in death [39,40]. The higher and earlier mortality observed at 300 mM compared to 200 mM further supports this conclusion. We used the 20-day mortality data to perform a log-logistic fit of the half-lethal concentration. The reason for selecting the 20-day data for the fit was that by this time, varieties Gongnong No.2 and Caoyuan No.1 had exhibited complete mortality, making this time point suitable for data fitting.

After ranking the LC_50_, we applied K-means clustering to categorize the varieties into salt-tolerant, moderate, and sensitive groups, thus providing better differentiation between salt-tolerant and sensitive alfalfa varieties. K-means clustering is a valuable tool for researchers to classify varieties, enhancing the focus and purpose of studies. For example, Atsa’am used this method in studies on West African cereals, and Mondal applied it in mango (*Mangifera indica* L.) research [41,42]. The salt-tolerant varieties largely included those already confirmed to be cultivable in saline–alkali regions of Northeast China, such as Zhaodong and Longmu801 [33,43]. To further investigate the salt tolerance mechanisms of alfalfa and identify salt tolerance genes, we selected Longmu801, a salt-tolerant variety, and WL168, a sensitive variety, as experimental varieties for further physiological measurements. As shown in Table S11, Longmu801 is a Chinese variety, while WL168 originates from the United States. Due to geographical differences and distinct breeding methods, the two varieties are expected to have significant genotypic differences [44]. Further research on these two varieties will provide more distinct and meaningful results.

In the variety screening section, we applied rigorous methods to identify cold-tolerant and salt-tolerant varieties. This not only establishes a solid theoretical foundation for our study but also provides a valuable reference for other researchers conducting alfalfa studies in the region. They can build upon our selected varieties for their research or introduce additional selection criteria to identify varieties that better align with their specific goals.

### 3.2. Interpretation of Salt Stress Effects on Alfalfa Growth and Physiology

To better understand the physiological responses and transcriptional regulatory mechanisms of alfalfa under salt stress, we employed 300 mM salt stress in this study. This concentration poses significant harm to alfalfa and induces clear physiological responses. To eliminate the inherent differences between the two varieties, we compared the relative values of the stress and control groups, which more accurately reflect the response of each variety to salt stress and highlight differences in salt tolerance.

First, both varieties showed slow or stagnant growth in terms of plant height. However, the relative plant height between the two varieties did not differ significantly. It is well known that plants reduce their growth rate and photosynthetic activity when subjected to stress as an adaptive response [45]. This suggests that both varieties experienced significant stress, leading to inhibited growth. For fresh weight and dry weight, both varieties exhibited an increasing trend over time, but the treated groups showed lower growth compared to the control groups. Notably, there was no significant difference in fresh weight between the two varieties on day 5. However, by day 7, a significant difference emerged. In contrast, dry weight did not show significant differences at any time point. This indicates that, under prolonged stress, Longmu801 has a better capacity for water regulation compared to WL168, allowing it to maintain its fresh weight and respond more effectively to salt stress.

Physiological indicators also showed significant differences under prolonged salt stress. In terms of photosynthetic pigments, chlorophyll a and total chlorophyll are important indicators of plant stress resistance [46,47] and are commonly studied in stress experiments with crops like maize [48], tomato [49], and rice [50]. In this study, both chlorophyll a, chlorophyll b, and total chlorophyll levels showed a decreasing trend under prolonged stress. Significant differences in total chlorophyll and chlorophyll a content were observed between the two varieties. Previous research suggests that chlorophyll a is more suitable for detecting abiotic stress [51], further highlighting the differences in salt tolerance between the two varieties. Hydrogen peroxide (H_2_O_2_) and malondialdehyde (MDA) are key indicators of oxidative stress in plants [52], and their levels are commonly used to assess the degree of stress [53,54,55]. In this experiment, the H_2_O_2_ and MDA levels of both varieties increased at 0, 1, 3, and 5 days. However, by day 7, WL168 had significantly higher levels compared to Longmu801, suggesting that Longmu801 has stronger salt tolerance under prolonged stress. Osmolytes are also key indicators of a plant’s stress resistance [56]. Proline, a typical osmotic regulator, plays a crucial role in osmotic adjustment under drought and salt stress [57]. In this study, proline levels showed significant changes. After one day of stress, both varieties began accumulating large amounts of proline, but Longmu801 exhibited significantly greater proline accumulation, indicating its stronger capacity to maintain cellular osmotic pressure under salt stress. Soluble sugars, which are critical for maintaining plant life activities, have been shown to increase significantly under abiotic stress due to a series of physiological activities [58]. In this study, the soluble sugar content of both varieties increased over time, but Longmu801 accumulated significantly more than WL168, and this difference became more pronounced as time progressed. This suggests that Longmu801 has stronger osmotic regulation capacity under salt stress. The higher accumulation of soluble sugars may also provide additional energy, helping Longmu801 better cope with the stress.

Additionally, antioxidant enzymes are important indicators of plant resistance to salt stress and reflect the plant’s ability to resist oxidative damage. Although WL168 had higher antioxidant enzyme activity before stress, the increase in antioxidant enzyme activity in Longmu801 under salt stress was significantly greater, suggesting that Longmu801 may have a stronger antioxidant capacity, allowing it to better cope with oxidative damage induced by salt stress. The antioxidant enzyme activity in both varieties exhibited an initial increase followed by a subsequent decline, which is a normal physiological response in plants. During the early stages of salt stress, alfalfa produces antioxidant enzymes to mitigate the stress, resulting in elevated enzyme activity. However, as the stress persists, the plant sustains damage, impairing its ability to maintain enzyme secretion, leading to a decline in enzyme activity. Nonetheless, the activity remains higher than that of the control (CK). If the stress continues until the plant dies, the enzyme activity would drop significantly below that of the CK. The differences in enzyme activity between the two varieties (Figure 8d−f) directly reflect their varying antioxidant capacities. This indicates that Longmu801 is better at sustaining antioxidant enzyme activity compared to WL168.

In this section, the growth and physiological indicators we measured are commonly used to evaluate the physiological state of plants under stress. For example, Feng et al. measured these indicators in a comparative study of salt tolerance in four buckwheat varieties and used a membership function to integrate the indicators for ranking [59]. Similarly, Rasel et al. utilized these physiological indicators to study different rice (*Oryza sativa* L.) genotypes under salt stress and applied clustering methods to screen salt-tolerant genotypes [60]. Quan et al. also employed these indicators in their research on salt tolerance among different alfalfa varieties and successfully obtained the desired results [61].

In terms of the salt stress experiment, what sets our research apart from others is that we used LC_50_ and clustering analysis to identify salt-tolerant and salt-sensitive varieties. We then selected specific varieties from these groups for further study, which significantly saved both experimental time and cost. It is also worth mentioning that in some variety studies, many researchers overlook the inherent differences between varieties and directly compare them, which we believe is not rigorous. In contrast, researchers such as Rasel [60] employed the susceptibility index (SI) for each variety, calculated as “(control value − salt treatment value)/control value × 100”. This approach is similar to the use of relative values in our study, as it helps eliminate the inherent differences between varieties when making comparisons, ensuring a more rigorous analysis. In summary, our findings indicate that the alfalfa varieties with differing salt tolerance exhibited similar trends in their growth and physiological response mechanisms. The differences between them were primarily reflected in the varying levels of the growth and physiological indicators.

Additionally, in this section, we used the relative values of physiological indicators from the two varieties to conduct grey correlation analysis to evaluate the key time points with the most significant differences. Grey correlation analysis has been widely used in various fields to optimize experiments [62,63,64]. In this study, day 7 was selected as the key time point for transcriptome sequencing, allowing for the identification of more critical differential genes.

### 3.3. Analysis of the Transcriptional Regulatory Mechanism of Alfalfa Under Salt Stress

Transcriptomic analysis revealed that, although the same reference genome, “Zhongmu No.4” was used, there were considerable differences in the DEGs between LM_ST vs. LM_CK and WL_ST vs. WL_CK under stress. This is due to the different gene expression levels among the varieties after salt stress. These differences can be attributed to the long-term independent breeding of the two varieties [44]. The results showed that WL had more DEGs than LM; however, WL exhibited poorer salt tolerance. This suggests that the additional DEGs may accelerate plant death, possibly because the increased genetic activity leads to faster energy metabolism and hastens the plant’s decline [65]. Alternatively, these genes may negatively regulate the plant’s stress tolerance, further contributing to its accelerated death [66]—a hypothesis that requires further validation.

To better compare the differences between LM and WL, we did not directly use LM_ST vs. WL_ST. Instead, we explored this aspect through LM_ST vs. LM_CK, WL_ST vs. WL_CK, and LM_ST vs. WL_ST comparisons. This approach effectively eliminates errors and biases caused by the inherent differences between the varieties. In this part, 2146 DEGs with similar expression trends were identified between the two alfalfa varieties. Among these, 1850 DEGs exhibited significant differential expression in Longmu801 compared to WL168, while 2679 DEGs showed significant differences in WL168 compared to Longmu801, likely reflecting genetic differences due to their respective evolutionary or breeding histories [44]. The enrichment of the 2146 DEGs in GO functions suggests that, under salt stress, alfalfa experiences extensive cellular activity in the nucleus, cytoplasm, plasma membrane, and membranes. ATP binding plays a crucial role in providing the energy required for these cellular activities [67], enabling the activation of stress-responsive transcription factors that, in turn, drive the expression of more stress-resistance genes [68]. Additionally, protein binding facilitates the formation of complexes to defend against salt stress, with these cellular activities being part of the plant’s response to oxidative stress.

The KEGG pathway enrichment analysis of these 2146 DEGs highlighted pathways such as phenylpropanoid biosynthesis (ko00940), protein processing in the endoplasmic reticulum (ko04141), and starch and sucrose metabolism (ko00500). The phenylpropanoid biosynthesis pathway is not only involved in the synthesis of lignin and flavonoids but also plays a critical role in gene regulation and DNA binding [69], providing a positive contribution to alfalfa’s defense against stress. The protein processing and starch and sucrose metabolism pathways further suggest that the plant requires more resistance-related or osmotic regulation proteins to combat stress. The increase in soluble sugar content observed in the physiological analysis is likely due to these metabolic activities. In addition to serving as a carbon source, sucrose also functions as a soluble sugar that regulates cellular osmotic pressure and helps maintain normal cellular activities.

Although 1850 DEGs were significantly different in Longmu801 and 2679 DEGs were significantly different in WL168, the GO functions and KEGG pathways of these DEGs were largely similar to the 2146 shared DEGs (as shown in Figures S2 and S3). This suggests that, despite genetic differences resulting from their evolutionary and breeding histories, both varieties rely on similar metabolic pathways and biological functions to resist salt stress. This contrasts with Lei’s study [70], where Lei posits that salt-tolerant varieties possess unique regulatory mechanisms, which contradicts our conclusions. In Lei’s research, the regulatory mechanisms refer more to the differences in transcription levels of salt-tolerant genes within salt-tolerant varieties compared to non-tolerant varieties, suggesting that the mechanisms are distinct. In our study, we prefer to use KEGG and GO analyses to explain the transcriptional regulatory mechanisms in alfalfa rather than focusing solely on a few specific genes. Our approach helps provide a more comprehensive explanation of the transcriptional regulatory mechanisms in alfalfa under salt stress. A conclusion consistent with Lei’s is that both studies agree that the differences between salt-tolerant and sensitive varieties arise from the expression levels of DEGs.

The conclusion we can draw from the transcriptomic analysis is that, although salt-tolerant and sensitive varieties exhibit similar transcriptional regulatory mechanisms in response to salt stress, the differences in DEGs between the varieties determine their variations in salt tolerance.

### 3.4. WGCNA-Based Hub Gene Mining and Functional Analysis

In this study, to better identify the key genes involved in salt stress resistance, we conducted WGCNA analysis using physiological indicators and transcriptomic data [71]. The WGCNA results identified seven modules (excluding the non-informative grey module), and we selected six modules that showed strong correlations with stress-resistant physiological traits for further analysis. Using WGCNA to identify hub genes is a common and effective approach. Zhu applied WGCNA in the study of salt-responsive hub genes in rice (*Oryza sativa* L.), identifying key transcription factors and genes within the relevant modules [72]. Similarly, Meng used WGCNA in their research on drought tolerance in Tartary buckwheat (*Fagopyrum tataricum* Gaertn.), discovering that HD-ZIP and MYB transcription factors may serve as key downstream regulators of drought resistance in buckwheat [73]. We conducted further research on the 30 hub genes identified and the 5 “abscisic acid and environmental stress-inducible protein” genes obtained from the transcriptomic analysis. To better understand the relationships among these 35 genes in alfalfa, we analyzed their correlations to gain insights into potential interactions, providing a more comprehensive understanding of their roles [74,75].

Further enrichment analysis of these genes revealed five hub genes directly related to salt stress. The results of the Mantel test showed a strong correlation between these five genes and physiological indicators. The Mantel test is a practical method for exploring the correlation between two different variables and has been widely applied in studies investigating the relationship between population genetics and environmental variables [76,77]. In this study, the Mantel test was used to analyze the correlation between the FPKM values of these five genes and physiological indicators, aiming to better explore the potential relationships between these genes and physiological traits.

Through WGCNA and further correlation analysis, this study not only identified hub genes but also explored the potential relationships among the hub genes and between the hub genes and physiological indicators. This deepens our understanding of these genes and provides a solid theoretical foundation for future research on them.

## 4. Materials and Methods

### 4.1. Preparation of Experimental Materials

Alfalfa (*Medicago sativa* L.) seeds were provided by the Forage Laboratory of Northeast Agricultural University. The variety and origin of the seeds are listed in Table S11. All seeds were sterilized with 15% sodium hypochlorite for 30 min and rinsed three times with sterile water. The seeds were then transferred to Petri dishes and incubated at a constant temperature of 25 °C in a plant incubator (GPL-250, Labotery, Tianjin, China) in darkness for one week, with the humidity maintained at 85%. After germination, the seedlings were transferred to 50-cell trays filled with pure vermiculite and irrigated with 1/5 Hoagland nutrient solution every two days. The seedlings were grown to the five-leaf stage before proceeding with subsequent experiments.

### 4.2. Variety Screening Process

#### 4.2.1. Cold Tolerance Screening Experiment

The alfalfa plants grown to the five-leaf stage were subjected to low-temperature stress at 4 °C. The experiment was conducted in the Grass Science Laboratory at Northeast Agricultural University. The specific procedure is as follows: First, the five-leaf stage alfalfa plants were placed in a plant incubator (GPL-250, Labotery, Tianjin, China) at room temperature (25 °C). The temperature was then gradually reduced by 2 °C per hour until it reached 4 °C, marking the beginning of the treatment period. Measurements of the plants were taken on day ten. Additionally, the light cycle in the incubator was set to 16 h of light and 8 h of darkness, with a light intensity of 7500 LX and humidity at 75%. For each variety, a treatment group was established at 4 °C, alongside a control group at room temperature (25 °C). Each group contained 50 plants, and three independent plants were randomly selected for measurement. The following indicators were measured: plant height, growth rate, fresh weight, dry weight, and biomass accumulation. Plant height was measured directly using a ruler. The growth rate was calculated by measuring the height on day 10, subtracting the initial height, and dividing by the height on day 1. Fresh weight was obtained by directly weighing the above-ground parts of the alfalfa. Dry weight was measured after drying the above-ground parts in a constant temperature drying oven (GZX-9023MBE, Boxun, Shanghai, China) at 65 °C until constant weight was achieved. The biomass accumulation was calculated as the fresh weight on day 10 minus the fresh weight on day 1. Cold tolerance was evaluated using the method by Xiang [78], where the relative values of various indicators were calculated to represent cold tolerance. Grey correlation analysis [79] was then used to evaluate cold tolerance, and the top 15 varieties were selected for the next stage of screening. The formula is as follows:

$$Cold\;tolerance\left(indicator\right)=\frac{Cold\;treatment\;group\;\left(indicator\right)}{Control\;group\;\left(indicator\right)}$$

#### 4.2.2. Salt Tolerance Screening Experiment

The selected varieties were subjected to salt tolerance screening using pot experiments in salt trays. To keep the vermiculite relatively dry while ensuring that the five-leaf stage alfalfa is not subjected to drought stress, we maintained the moisture needs of the alfalfa primarily by spraying water daily for about five days at the beginning of the experiment rather than using irrigation. We placed the trays containing five-leaf stage alfalfa, with carefully controlled vermiculite moisture into large trays filled with salt solution, allowing the vermiculite to fully absorb the salt solution. Starting on the first day after treatment, we recorded the number of dead plants under different NaCl concentrations (0, 100 mM, 200 mM, 300 mM). Each variety was established with three independent replicates, each consisting of fifty individual plants. A log-logistic half-lethal concentration fitting was then performed [80], followed by K-means clustering analysis to identify salt-tolerant and salt-sensitive varieties [81]. To ensure the accuracy of the salt treatments, the salt concentration, salt content, and electrical conductivity of the vermiculite suspension in the trays treated with different salt concentrations were measured according to the methods outlined in “Soil Agrochemical Analysis” [82]. The results are shown in Supplementary Figure S6. A correlation analysis between the salt concentration of the vermiculite extract and the salt treatment solution showed R^2^ = 0.99, indicating that the salt concentration in the vermiculite was consistent with that of the treatment solution.

### 4.3. Measurement of Growth and Physiological Indicators Under Salt Stress

A salt stress experiment was conducted on salt-tolerant and salt-sensitive varieties using 300 mM NaCl. As mentioned in Section 4.2.2 regarding the salt stress experiment, we employed the method of pot experiments in salt trays for this experiment. Each variety had a salt treatment group and a control group (the control group used distilled water instead of salt solution). Each group contained 50 alfalfa plants, and three independent plants were randomly selected from these 50 for measurement. Growth indicators were measured at 0, 3, and 7 days, and physiological indicators were measured at 0, 1, 3, 5, and 7 days. Grey correlation analysis was performed at different time points to identify the optimal time for transcriptomic analysis.

Growth indicators included measuring plant height using a ruler and assessing fresh and dry weights through a weighing method. Fresh weight was measured by taking the above-ground parts of the alfalfa and directly weighing them. Dry weight was obtained by placing the weighed fresh samples in a constant temperature drying oven (GZX-9023MBE, Boxun, Shanghai, China) at 65 °C until a constant weight was achieved, after which the weight was measured.

The physiological indicators were measured using kits purchased from Suzhou Keming Biological Co., Ltd., Suzhou, China (http://www.cominbio.com/index.html (accessed on 10 September 2023)). The above-ground parts of the alfalfa were used for sample grinding, followed by homogenization for measurement. Specifically, proline content was measured using the sulfosalicylic acid method, malondialdehyde (MDA) and soluble sugars were measured using the thiobarbituric acid method, chlorophyll was measured using the anhydrous ethanol extraction method, and hydrogen peroxide was extracted with acetone. Superoxide dismutase (SOD), peroxidase (POD), and catalase (CAT) activities were measured using spectrophotometry (N5000Plus, Yoke Instrument, Shanghai, China) [83]. Grey correlation analysis [79] was then used to select the key time point for transcriptomic sequencing.

### 4.4. Transcriptomic Analysis

Sampling was performed at the key time points determined from the salt stress experiment, followed by transcriptomic analysis. The specific steps are as follows: After 7 days of salt stress and control treatments, samples were collected from Longmu801 and WL168, primarily taking the above-ground parts of the alfalfa, which were then ground and homogenized. For each variety, one group of control treatment and one group of salt treatment were collected, totaling four groups. Four biological replicates were collected for each treatment and sent to LC-Bio Technologies Co., Ltd. (Hangzhou, China) for testing under dry ice conditions, with approximately 0.5 g of sample per replicate.

Total RNA was isolated from tissue samples using TRIzol reagent (Invitrogen, Waltham, MA, USA) according to the manufacturer’s protocol. RNA quantity and purity were assessed using a NanoDrop ND-1000 spectrophotometer (ThermoFisher, Waltham, MA, USA), and RNA integrity was evaluated using the Agilent 2100 Bioanalyzer system (Agilent, Santa Clara, CA, USA). Samples with an RNA integrity number (RIN) greater than 7.0 were used for downstream analysis, primarily assessed using the RNA 6000 Nano LabChip Kit (Agilent, Santa Clara, CA, USA, 5067-1511). Polyadenylated mRNA was enriched using PolyA and Oligo(dT) magnetic beads. The mRNA was fragmented using divalent cations, and first-strand cDNA was synthesized using reverse transcriptase. Double-stranded cDNA was generated using DNA polymerase I and RNase H. The ends of the double-stranded cDNA were blunted, an A-base was added, and adapters were ligated. The resulting fragments (~300 bp) were purified and amplified by PCR. Libraries were sequenced on the Illumina HiSeq 4000 platform to generate 150 bp paired-end reads.

The raw data were further filtered by Cutadapt (https://cutadapt.readthedocs.io/en/stable (accessed on 11 December 2023), version 1.9). After deleting low-quality and repeated sequences, high-quality clean reads were produced. The clean data were then compared to the alfalfa genome (https://modms.lzu.edu.cn/alfalfa/browse/genome/ZM-4, https://modms.lzu.edu.cn/alfalfa/browse/genome/ZMNO, https://modms.lzu.edu.cn/alfalfa/browse/genome/XJDY, accessed on 11 December 2023) [27,84] using HISAT2 (hierarchical indexing for spliced alignment of transcripts) (https://daehwankimlab.github.io/hisat2 (accessed on 11 December 2023), version 2.0.4) to obtain the BAM file. The initial assembly of transcripts for each sample was accomplished using StringTie (http://ccb.jhu.edu/software/stringtie/ (accessed on 11 December 2023), version 2.1.6) with default parameters, and the mapped reads were merged to reconstruct a comprehensive transcriptome. Subsequently, gffcompare software (http://ccb.jhu.edu/software/stringtie/gffcompare.shtml (accessed on 11 December 2023), version 0.9.8) was employed to compare the assembled transcripts with the reference annotation, generating the final assembly annotation. After producing the final transcriptome, StringTie and ballgown (http://www.bioconductor.org/packages/release/bioc/html/ballgown.html (accessed on 11 December 2023), version 2.12.0) were used to estimate expression levels of all transcripts and calculate FPKM (fragments per kilobase of transcript per million mapped reads) values for mRNAs, with ballgown facilitating the FPKM quantification file input [85,86,87]. The R package edgeR was used to assess the statistically significant differences between samples. Differentially expressed genes (DEGs) were identified as those with a multiple-fold change of more than 2 (upregulate) or less than 0.5 (downregulate) and a *p*-value < 0.05. The biological functions and signaling pathways of DEGs were classified using GO (Gene Ontology) and KEGG (Kyoto Encyclopedia of Genes and Genomes) enrichment analyses.

### 4.5. qRT-PCR Validation Procedure

The RNA used for qPCR was extracted from samples collected after 7 days of salt treatment, ensuring consistency with the samples used for transcriptomic analysis. The RNA extraction was carried out using the Ultrapure RNA Kit (Cowin Biotech, Taizhou, China) from Cowin Biotech. cDNA was synthesized using the HiScript II Q RT SuperMix for qPCR (+gDNA wiper) kit (Vazyme Biotech, Nanjing, China) from Vazyme Biotech. This was followed by qPCR analysis.

For qPCR, GAPDH was used as the reference gene [88], and the primers for validating the DEGs from the transcriptome data can be found in Supplementary Table S12. The qPCR instrument used for quantification was the q225 model (q225, Kubo Technology, Beijing, China) from Kubo Technology. The qPCR assays followed the three-step protocol, using the 2× SYBR Green qPCR Master Mix II (Universal) (Seven Biotech, Beijing, China). The detailed reaction protocol is provided in Table S13.

### 4.6. Gene Co-Expression Network Analysis (WGCNA)

In this study, we performed WGCNA using the WGCNA cloud tool (https://www.omicstudio.cn/analysis, accessed on 27 December 2023) [89] provided by the LC-Bio cloud platform. The specific procedures are as follows:

First, we compiled the FPKM values of all detected genes from the transcriptomic data of LM_CK, LM_ST, WL_CK, and WL_ST samples, with each group comprising four biological replicates, resulting in a total of 16 datasets. This sample size meets the requirement for conducting WGCNA (a minimum of 15 samples). We subsequently filtered out genes with an expression level below 2, yielding a final count of 32,684 genes.

Next, we conducted a clustering analysis of the different samples to eliminate any outlier data points, followed by further WGCNA analysis. We then determined the soft threshold, setting the platform threshold to 0.85 and the soft threshold to 1. To enhance the biological significance of our findings, we employed the topological overlap measure (TOM) to evaluate the degree of association between genes, taking into account not only the interactions between pairs of genes but also their connections with other genes in the network. We also calculated the correlation between modules and samples, generating a correlation heatmap to identify modules most closely associated with the samples. Additionally, GO and KEGG enrichment analyses were performed to further elucidate the functional roles of genes within the modules. Finally, hub genes within different modules were identified using Cytoscape (v3.9.1) in conjunction with the MCODE plugin. Finally, key genes were identified based on degree values within each module using Cytoscape (v3.9.1) and the MCODE plugin [90].

### 4.7. Hub Gene Correlation Analysis and Functional Study

The expression levels of hub genes under different treatments were analyzed using the LC-Bio cloud platform (https://www.omicstudio.cn/tool, accessed on 10 March 2024). Specifically, the FPKM values of hub genes in LM_CK, LM_ST, WL_CK, and WL_ST were calculated and further analyzed using the platform’s correlation analysis tool (https://www.omicstudio.cn/tool/62, accessed on 10 March 2024). The results were visualized using the platform’s visualization tool (https://www.omicstudio.cn/tool/64, accessed on 10 March 2024) [89].

Additionally, the GO functions and KEGG pathways of hub genes were visualized using a Sankey diagram on the same platform (https://www.omicstudio.cn/tool/155, accessed on 10 March 2024). To evaluate the correlation between salt stress-related genes’ FPKM and physiological indicators across different varieties, a Mantel test was performed (https://www.omicstudio.cn/tool/109, accessed on 10 March 2024) [91].

## 5. Conclusions

In this study, Longmu801 was identified as a salt-tolerant alfalfa variety, while WL168 was identified as a salt-sensitive variety. The two varieties exhibited the same growth and physiological response mechanisms, with their differences primarily reflected in the varying levels of the indicators. When subjected to salt stress, Longmu801 maintained higher levels of osmolytes (proline and soluble sugars), antioxidant enzymes (SOD, POD, and CAT), chlorophyll, and fresh weight compared to WL168, along with lower levels of oxidative stress markers (malondialdehyde and hydrogen peroxide). Transcriptomic analysis revealed significant differences in DEGs between the two varieties; however, the enriched GO functions (such as oxidative stress, nucleus, protein binding, etc.) and KEGG pathways (including phenylpropanoid biosynthesis, protein processing in the endoplasmic reticulum, and starch and sucrose metabolism, etc.) were remarkably similar. This suggests that both varieties share analogous transcriptional regulatory mechanisms in response to salt stress. Furthermore, through weighted gene co-expression network analysis (WGCNA), five hub genes directly associated with salt response were identified: *Msa085011*, *Msa0605650*, *Msa0397400*, *Msa1258740*, and *Msa0958830*.

In summary, the two varieties exhibit similar growth and physiological response mechanisms, as well as analogous transcriptional regulatory mechanisms under salt stress. Their differences in salt tolerance are primarily attributed to variations in the levels of specific indicators and the expression levels of DEGs. This will provide a theoretical basis and research foundation for breeding salt-tolerant alfalfa.

## Data Availability

The RNA-seq datasets generated during the current study have been submitted to the NCBI Sequence Read Archive under the accession number PRJNA1110792 (https://dataview.ncbi.nlm.nih.gov/object/PRJNA1110792. Release date: 31 October 2024).

## Supplementary Materials

The following supporting information can be downloaded at: https://www.mdpi.com/article/10.3390/plants13223141/s1. Figure S1. Mortality rates of different alfalfa varieties; Figure S2. Analysis of 1850 DEGs; Figure S3. Analysis of 2679 DEGs; Figure S4. qRT-PCR validation of transcriptomic data; Figure S5. Functional study of genes in different WGCNA modules; Figure S6. Verification of salt concentrations in the salt stress experiment; Table S1. Effects of cold stress on the plant height of different alfalfa varieties.; Table S2. Effects of cold stress on the growth rate of different alfalfa Varieties; Table S3. Effects of cold stress on the fresh weight of different alfalfa varieties; Table S4. Effects of cold stress on the increase in biomass of different alfalfa varieties; Table S5. Effects of cold stress on the dry weight of different alfalfa varieties; Table S6. Effects of cold stress on relative growth indicators of different alfalfa varieties; Table S7. Unweighted and weighted grey correlation of different alfalfa varieties; Table S8. Statistical table for assessment of sample sequencing data; Table S9. Comparative analysis of different alfalfa genomes; Table S10. Comparison table between sample and reference genome; Table S11. Origin and location of alfalfa varieties; Table S12. Primers of five selected DEGs for qRT-PCR analysis; Table S13. qRT-PCR reaction protocol.
