# Supplementary material for: Unveiling Salt Tolerance Mechanisms and Hub Genes in Alfalfa (Medicago sativa L.) Through Transcriptomic and WGCNA Analysis
Source: Plants (Basel). 2024 Nov 8;13(22):3141. doi: 10.3390/plants13223141 (PMC11597802; doi:10.3390/plants13223141)
Supplement: Supplementary file 1 [file plants-13-03141-s001.zip › Figure S1~S6.zip!/Figure S2.pdf]

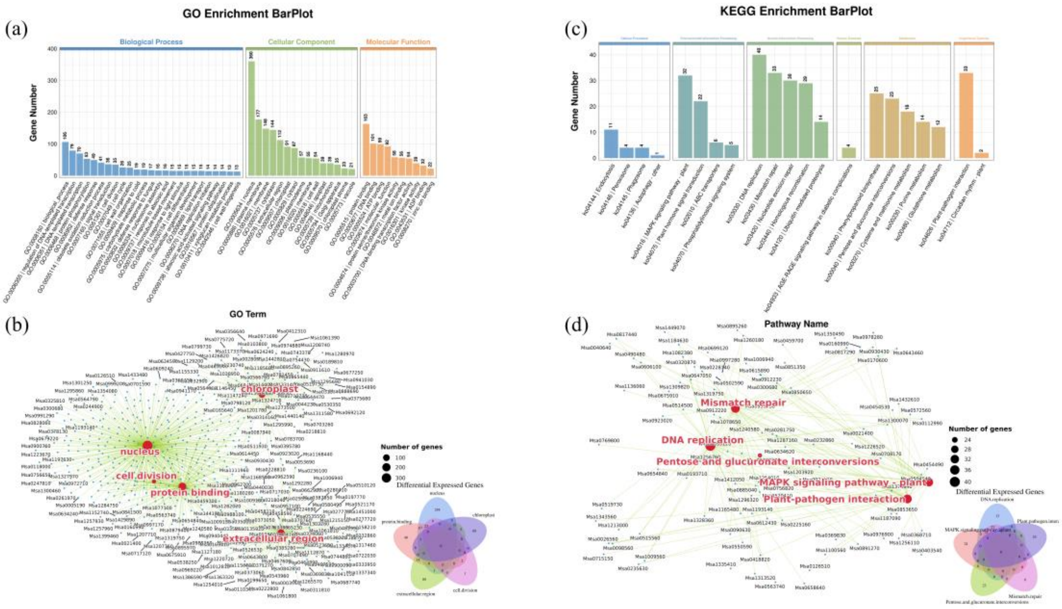

Figure S2. Analysis of 1850 DEGs. (a) GO Functions of 1850 DEGs. (b) the Relationship between GO Functions and DEGs. (c) KEGG Pathways of 1850 DEGs. (d) the Relationship between KEGG Pathways and DEGs.
