# Supplementary material for: Unveiling Salt Tolerance Mechanisms and Hub Genes in Alfalfa (Medicago sativa L.) Through Transcriptomic and WGCNA Analysis
Source: Plants (Basel). 2024 Nov 8;13(22):3141. doi: 10.3390/plants13223141 (PMC11597802; doi:10.3390/plants13223141)
Supplement: Supplementary file 1 [file plants-13-03141-s001.zip › Figure S1~S6.zip!/Figure S3.pdf]

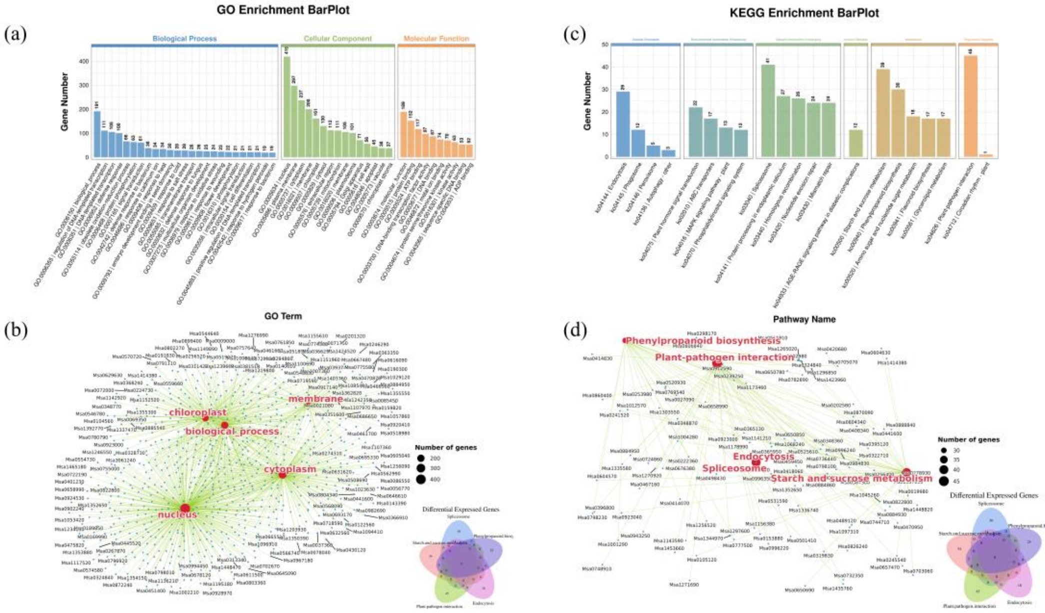

Figure S3. Analysis of 2679 DEGs. (a) GO Functions of 2679 DEGs. (b) the Relationship between GO Functions and DEGs. (c) KEGG Pathways of 2679 DEGs. (d) the Relationship between KEGG Pathways and DEGs.
