# Supplementary material for: Unveiling Salt Tolerance Mechanisms and Hub Genes in Alfalfa (Medicago sativa L.) Through Transcriptomic and WGCNA Analysis
Source: Plants (Basel). 2024 Nov 8;13(22):3141. doi: 10.3390/plants13223141 (PMC11597802; doi:10.3390/plants13223141)
Supplement: Supplementary file 1 [file plants-13-03141-s001.zip › Figure S1~S6.zip!/Figure S4.pdf]

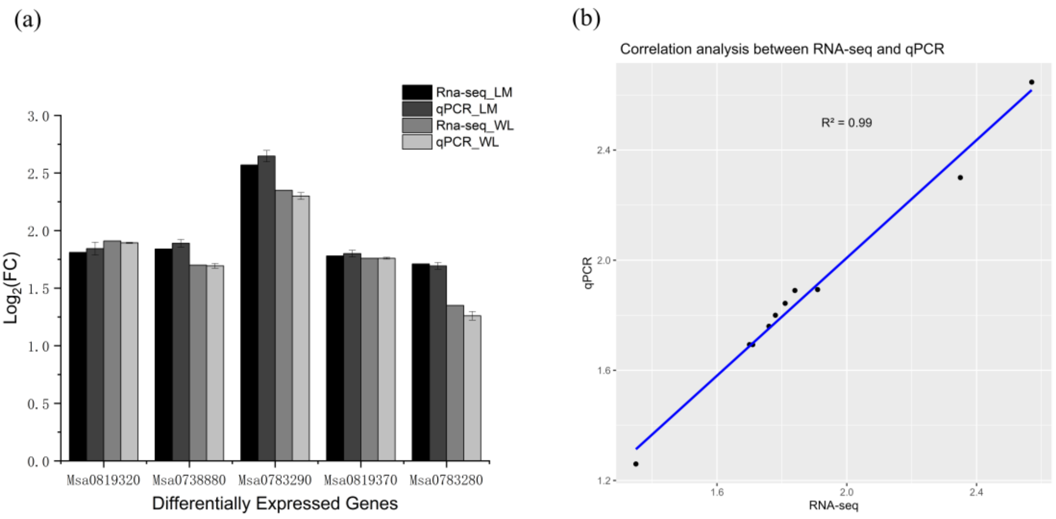


**Figure S4.** qRT-PCR validation of transcriptomic data. (a) RT-PCR validation. (b) Correlation analysis between transcriptomic data and qRT-PCR results.
