# Supplementary material for: Unveiling Salt Tolerance Mechanisms and Hub Genes in Alfalfa (Medicago sativa L.) Through Transcriptomic and WGCNA Analysis
Source: Plants (Basel). 2024 Nov 8;13(22):3141. doi: 10.3390/plants13223141 (PMC11597802; doi:10.3390/plants13223141)
Supplement: Supplementary file 1 [file plants-13-03141-s001.zip › Figure S1~S6.zip!/Figure S6.pdf]

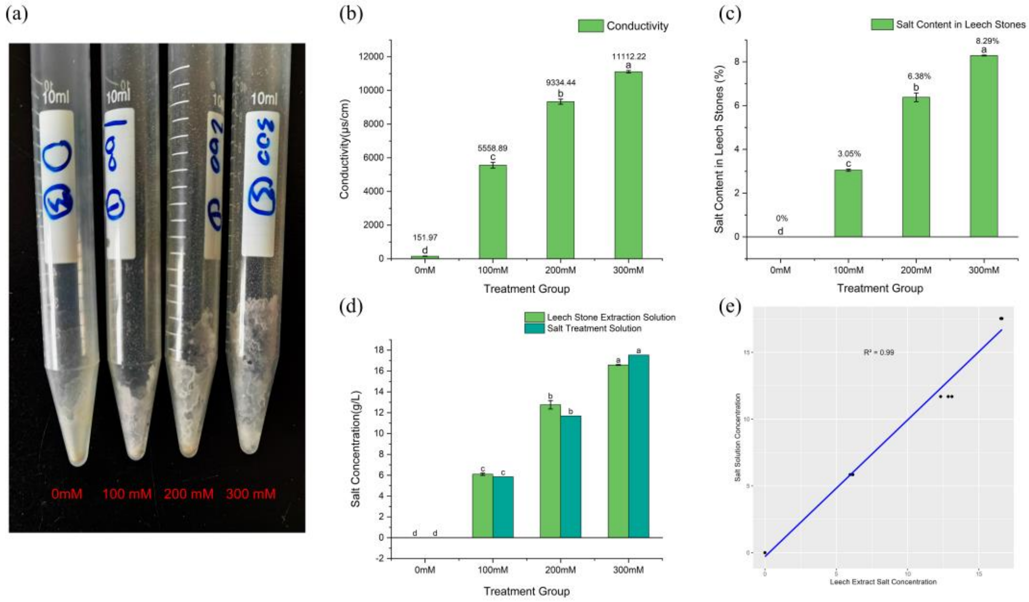

Figure S6. Verification of salt concentrations in the salt stress experiment. (a) Image of Salt Content in Vermiculite: The NaCl content remaining in the centrifuge tube after drying vermiculite suspensions treated with different salt concentrations. (b) Electrical conductivity of the vermiculite suspension. (c) Salt content in vermiculite at different salt concentrations. (d) Salt concentration in the salt treatment solution and in the vermiculite extract. (e) Correlation analysis between the salt concentration of the vermiculite extract and the salt treatment solution.
