# Supplementary material for: Unveiling Salt Tolerance Mechanisms and Hub Genes in Alfalfa (Medicago sativa L.) Through Transcriptomic and WGCNA Analysis
Source: Plants (Basel). 2024 Nov 8;13(22):3141. doi: 10.3390/plants13223141 (PMC11597802; doi:10.3390/plants13223141)
Supplement: Supplementary file 1 [file plants-13-03141-s001.zip › Table S1~S13.zip!/Table S1.pdf]

Table S1. Effects of Cold Stress on the Plant Height of Different Alfalfa Varieties.

	Plant Height (cm)			
	0d		10d	
	4°C	CK	4°C	CK
Juneng 401	6.10 ± 0.30 a	6.23 ± 0.40 a	7.80 ± 0.44 b	9.17 ± 0.42 c
Jinneng 5010	7.20 ± 0.35 a	7.47 ± 0.21 a	7.43 ± 0.40 a	9.40 ± 0.10 b
Juneng 551	6.30 ± 0.26 a	6.50 ± 0.30 a	7.77 ± 0.21 b	10.30 ± 0.20 c
Womu No. 1	5.87 ± 0.40 a	6.20 ± 0.44 a	7.03 ± 0.06 b	10.23 ± 0.93 c
Nongjing No. 1	6.80 ± 0.44 a	6.83 ± 0.45 a	10.60 ± 0.36 b	11.83 ± 0.47 c
SR4030	7.30 ± 0.46 a	7.23 ± 0.31 ab	8.37 ± 0.40 b	11.27 ± 0.25 c
MF4015	9.27 ± 0.25 a	9.57 ± 0.32 a	9.60 ± 0.10 a	12.83 ± 1.61 b
Juneng No. 1	8.27 ± 0.06 a	8.53 ± 0.71 a	8.63 ± 0.15 a	9.20 ± 0.78 b
Instar	7.80 ± 0.30 a	8.10 ± 0.61 a	8.20 ± 0.17 a	9.23 ± 0.64 b
Longmu 801	8.90 ± 0.17 a	9.20 ± 0.56 a	9.33 ± 0.29 a	10.20 ± 0.66 b
Golden Queen	7.47 ± 0.55 a	7.90 ± 1.05 a	8.00 ± 0.44 a	9.30 ± 0.69 b
Longmu 806	7.07 ± 0.64 a	7.43 ± 0.81 a	9.07 ± 0.21 b	9.87 ± 0.40 c
Zhaodong	7.57 ± 0.21 a	7.50 ± 0.10 a	9.73 ± 0.25 b	9.87 ± 0.40 b
Longmu 803	8.63 ± 0.35 a	8.53 ± 0.49 a	9.53 ± 0.83 b	10.97 ± 0.25 c
Salt Star	7.37 ± 0.40 a	7.73 ± 0.25 a	8.10 ± 0.17 b	10.23 ± 0.46 c
Spade	6.77 ± 0.25 a	6.73 ± 0.59 a	7.73 ± 0.49 a	11.17 ± 0.76 b
Gongnong No.1	8.10 ± 0.26 a	8.33 ± 0.31 ab	8.83 ± 0.15 bc	9.33 ± 0.59 c
Saiyuan	7.00 ± 0.10 a	6.90 ± 0.26 a	7.77 ± 0.25 b	10.10 ± 0.56 c
WL298 HQ	6.83 ± 0.38 a	7.30 ± 0.30 a	9.43 ± 0.51 b	12.70 ± 1.84 c
Knight 2	7.90 ± 0.46 a	7.83 ± 0.75 a	9.67 ± 0.15 b	11.60 ± 0.79 c
MF4020	8.50 ± 0.20 a	8.57 ± 0.31 a	9.93 ± 0.60 b	11.33 ± 0.15 c
Caoyuan No.2	8.83 ± 0.65 a	8.73 ± 0.38 a	9.77 ± 0.25 b	12.10 ± 0.69 c
Kangsai 2	8.67 ± 0.35 a	8.47 ± 0.15 a	9.20 ± 0.26 b	10.20 ± 0.36 c
Zhongmu No.1	8.40 ± 0.10 a	8.33 ± 0.45 a	8.97 ± 0.35 a	12.00 ± 1.32 b
Fudi	8.90 ± 0.20 a	8.73 ± 0.25 a	9.97 ± 0.64 b	11.00 ± 0.50 c
Challenger	8.53 ± 0.25 a	8.70 ± 0.20 a	9.47 ± 0.15 b	11.00 ± 0.50 c
Russia	8.27 ± 0.25 a	8.27 ± 0.15 a	8.83 ± 0.15 b	10.20 ± 0.85 c
Crown	8.07 ± 0.21 a	8.10 ± 0.26 a	8.90 ± 0.36 b	9.43 ± 0.51 b
Caoyuan No.3	7.43 ± 0.12 a	7.43 ± 0.15 a	8.63 ± 0.15 b	9.93 ± 1.21 c
Baimu 202	6.70 ± 0.10 a	6.73 ± 0.32 a	7.03 ± 0.25 a	7.77 ± 0.25 b
Juneng No.2	7.80 ± 0.50 a	7.83 ± 0.31 a	8.93 ± 0.25 b	10.40 ± 0.66 c
Juneng 201	6.50 ± 0.30 a	6.70 ± 0.26 a	8.13 ± 0.47 ab	9.70 ± 0.70 c
Kateruo	7.77 ± 0.25 a	7.70 ± 0.36 a	8.83 ± 0.61 b	10.43 ± 0.51 c
Dryland	7.67 ± 0.31 a	7.63 ± 0.31 a	8.27 ± 0.25 ab	9.93 ± 1.37 b
Gongnong No.2	7.73 ± 0.25 a	7.70 ± 0.36 a	10.50 ± 0.62 b	11.47 ± 0.50 c
Caoyuan No.1	7.77 ± 0.31 a	7.77 ± 0.45 a	10.20 ± 0.26 b	10.27 ± 0.64 b
Weiner	7.63 ± 0.42 a	7.83 ± 0.35 a	8.63 ± 0.65 a	10.67 ± 0.76 b
WL168 HQ	7.03 ± 0.25 a	7.17 ± 0.21 a	7.93 ± 0.51 b	9.30 ± 0.26 c
DY2006	7.40 ± 0.10 a	7.33 ± 0.21 a	8.10 ± 0.36 b	10.67 ± 0.72 bc
Weiner	7.83 ± 0.15 a	7.90 ± 0.20 a	8.47 ± 0.25 ab	8.97 ± 0.47 b
Dongnong No.1	7.23 ± 0.25 a	7.30 ± 0.10 a	8.03 ± 0.25 b	8.53 ± 0.40 b

Note: 4°C indicates cold treatment, while CK represents normal conditions.
