# Supplementary material for: Unveiling Salt Tolerance Mechanisms and Hub Genes in Alfalfa (Medicago sativa L.) Through Transcriptomic and WGCNA Analysis
Source: Plants (Basel). 2024 Nov 8;13(22):3141. doi: 10.3390/plants13223141 (PMC11597802; doi:10.3390/plants13223141)
Supplement: Supplementary file 1 [file plants-13-03141-s001.zip › Table S1~S13.zip!/Table S10.pdf]

Table S10. Comparison Table Between Sample and Reference Genome.

Sample	Valid reads	Mapped reads (%)	Unique Mapped %	PE Mapped %	Non-splice %	Splice %
LM_CK1	35930510	30258802(84.21%)	36.98%	72.23%	23.12%	13.86%
LM_CK2	39775910	34251755(86.11%)	37.63%	74.27%	23.53%	14.10%
LM_CK3	41592130	36305154(87.29%)	38.02%	75.44%	23.78%	14.25%
LM_CK4	39352780	35381051(89.91%)	39.54%	78.28%	24.69%	14.85%
LM_ST1	37845170	32332346(85.43%)	37.39%	74.45%	22.46%	14.93%
LM_ST2	37859570	32593263(86.09%)	37.62%	74.91%	22.62%	15.00%
LM_ST3	41421292	35654015(86.08%)	37.57%	75.11%	22.65%	14.93%
LM_ST4	39128456	33775753(86.32%)	37.37%	74.16%	22.55%	14.82%
WL_CK1	36722816	31671561(86.24%)	37.74%	73.27%	23.93%	13.81%
WL_CK2	35987452	32016524(88.97%)	38.75%	75.23%	24.66%	14.09%
WL_CK3	38263098	34413892(89.94%)	39.15%	75.93%	25.04%	14.10%
WL_CK4	35845360	32179540(89.77%)	39.19%	76.08%	25.01%	14.18%
WL_ST1	38708232	34398556(88.87%)	38.99%	75.01%	24.48%	14.51%
WL_ST2	34829836	30935764(88.82%)	38.85%	74.93%	24.38%	14.47%
WL_ST3	35441784	31659117(89.33%)	39.11%	75.26%	24.60%	14.51%
WL_ST4	33821714	28662951(84.75%)	37.21%	72.21%	23.31%	13.90%
