# Supplementary material for: Unveiling Salt Tolerance Mechanisms and Hub Genes in Alfalfa (Medicago sativa L.) Through Transcriptomic and WGCNA Analysis
Source: Plants (Basel). 2024 Nov 8;13(22):3141. doi: 10.3390/plants13223141 (PMC11597802; doi:10.3390/plants13223141)
Supplement: Supplementary file 1 [file plants-13-03141-s001.zip › Table S1~S13.zip!/Table S11.pdf]

**Table S11**. Origin and Location of Alfalfa Varieties.

| No. | Variety Name | Source | Location | No. | Variety Name | Source | Location |
|---|---|---|---|---|---|---|---|
| 1 | Baimu 202 | Baiqingyuan Co., Ltd. | USA | 22 | Kangsai 2 | Baiqingyuan Co., Ltd. | China |
| 2 | Caoyuan No. 1 | Inner Mongolia Agricultural University | China | 23 | Kateruo | Baiqingyuan Co., Ltd. | China |
| 3 | Caoyuan No. 2 | Inner Mongolia Agricultural University | China | 24 | Longmu 801 | Heilongjiang Institute of Animal Husbandry | China |
| 4 | Caoyuan No. 3 | Inner Mongolia Agricultural University | China | 25 | Longmu 803 | Heilongjiang Institute of Animal Husbandry | China |
| 5 | Challenger | Baiqingyuan Co., Ltd. | China | 26 | Longmu 806 | Heilongjiang Institute of Animal Husbandry | China |
| 6 | Crown | Beijing Clover Co., Ltd. | USA | 27 | MF 4015 | Beijing Clover Co., Ltd. | Canada |
| 7 | Dongnong No. 1 | Northeast Agricultural University | China | 28 | MF 4020 | Beijing Clover Co., Ltd. | Canada |
| 8 | DY 2006 | Northeast Agricultural University | China | 29 | Nongjing No. 1 | National Germplasm Center for Cold Region Crops & Soybeans | China |
| 9 | Dryland | Beijing Clover Co., Ltd. | USA | 30 | Reindeer | Heilongjiang Institute of Animal Husbandry | Russia |
| 10 | Fudi | Baiqingyuan Co., Ltd. | China | 31 | Russia | Heilongjiang Institute of Animal Husbandry | Russia |
| 11 | Gongnong No. 1 | Jilin Academy of Agricultural Sciences | China | 32 | SR 4030 | Beijing Clover Co., Ltd. | Canada |
| 12 | Gongnong No. 2 | Jilin Academy of Agricultural Sciences | China | 33 | Salt Star | Beijing Clover Co., Ltd. | China |
| 13 | Golden | Beijing | USA | 34 | Saiyuan | Baiqingyuan | China |

| | | | | | | | |
|---|---|---|---|---|---|---|---|
| | Queen | Clover Co., Ltd. | | | | Co., Ltd. | |
| 14 | Instar | Beijing Clover Co., Ltd. | USA | 35 | Spade | Beijing Clover Co., Ltd. | USA |
| 15 | Juneng No. 1 | Beijing Clover Co., Ltd. | USA | 36 | WL168 HQ | Beijing Clover Co., Ltd. | USA |
| 16 | Juneng No. 2 | Beijing Clover Co., Ltd. | USA | 37 | WL298 HQ | Beijing Clover Co., Ltd. | USA |
| 17 | Juneng 201 | Beijing Clover Co., Ltd. | USA | 38 | Weiner | Beijing Clover Co., Ltd. | USA |
| 18 | Juneng 401 | Beijing Clover Co., Ltd. | USA | 39 | Womu No. 1 | Beijing Clover Co., Ltd. | USA |
| 19 | Juneng 551 | Beijing Clover Co., Ltd. | USA | 40 | Zhongmu No. 1 | Institute of Animal Husbandry, Chinese Academy of Agricultural Sciences | China |
| 20 | Jinneng 5010 | Northeast Agricultural University | China | 41 | Zhaodong | Northeast Agricultural University | China |
| 21 | Knight 2 | Baiqingyuan Co., Ltd. | China | | | | |
