# Supplementary material for: Unveiling Salt Tolerance Mechanisms and Hub Genes in Alfalfa (Medicago sativa L.) Through Transcriptomic and WGCNA Analysis
Source: Plants (Basel). 2024 Nov 8;13(22):3141. doi: 10.3390/plants13223141 (PMC11597802; doi:10.3390/plants13223141)
Supplement: Supplementary file 1 [file plants-13-03141-s001.zip › Table S1~S13.zip!/Table S12.pdf]

Table S12. Primers of 5 selected DEGs for qRT-PCR analysis.

Gene id	Primer(5′-3′)	
Msa0819320	Forward	ACCTCTGATGCCAAAAAGGAAG
	Reverse	CCTCCACCATTGTAACCACC
Msa0859490	Forward	GCCAAATATGGTGGTGGTGG
	Reverse	CAGTTCTGAGTTTTGTCCTCAGC
Msa0859510	Forward	CTGATGCCAAAAAGGGTGGTT
	Reverse	ATACACAGCAACTCCTCCACC
Msa0783290	Forward	TTCTTGTGTCAGCTAGGGACT
	Reverse	CACCATTGTGGTAACCGCC
Msa0819370	Forward	CCTCAGAAGTCTCAGCTAGGG
	Reverse	CACCATTGTAACCACCACCG
GAPDH	Forward	GGCTGCCATCAAGGAGGAAT
	Reverse	TCCAAGCTCAGCCTCATCAAG
