# Supplementary material for: Unveiling Salt Tolerance Mechanisms and Hub Genes in Alfalfa (Medicago sativa L.) Through Transcriptomic and WGCNA Analysis
Source: Plants (Basel). 2024 Nov 8;13(22):3141. doi: 10.3390/plants13223141 (PMC11597802; doi:10.3390/plants13223141)
Supplement: Supplementary file 1 [file plants-13-03141-s001.zip › Table S1~S13.zip!/Table S13.pdf]

Table S13. qRT-PCR reaction protocol.

Steps	Temperature	Reaction time	
Pre-denaturation	95℃	30 second	
Denaturation	95℃	10~20 second	35~45 cycles
Annealing	60℃	10~20 second	
Extension	72℃	20~30 second	
Dissociation Curve Analysis		According to the instrument's recommended protocol.	
