# Supplementary material for: Unveiling Salt Tolerance Mechanisms and Hub Genes in Alfalfa (Medicago sativa L.) Through Transcriptomic and WGCNA Analysis
Source: Plants (Basel). 2024 Nov 8;13(22):3141. doi: 10.3390/plants13223141 (PMC11597802; doi:10.3390/plants13223141)
Supplement: Supplementary file 1 [file plants-13-03141-s001.zip › Table S1~S13.zip!/Table S2.pdf]

Table S2. Effects of Cold Stress on the Growth Rate of Different Alfalfa Varieties.

	Growth Rate (cm/d) 0-10d	
	4℃	CK
Juneng 401	0.17 ± 0.07 a	0.29 ± 0.08 b
Jinneng 5010	0.02 ± 0.02 a	0.19 ± 0.01 b
Juneng 551	0.15 ± 0.02 a	0.38 ± 0.05 b
Womu No. 1	0.12 ± 0.04 a	0.40 ± 0.11 b
Nongjing No. 1	0.38 ± 0.05 a	0.50 ± 0.04 b
SR4030	0.11 ± 0.02 a	0.40 ± 0.06 b
MF4015	0.03 ± 0.03 a	0.33 ± 0.13 b
Juneng No. 1	0.04 ± 0.01 a	0.07 ± 0.05 b
Instar	0.04 ± 0.02 a	0.11 ± 0.01 b
Longmu801	0.04 ± 0.01 a	0.10 ± 0.05 b
Golden Queen	0.05 ± 0.03 a	0.14 ± 0.13 b
Longmu 806	0.20 ± 0.04 a	0.24 ± 0.11 a
Zhaodong	0.22 ± 0.02 a	0.24 ± 0.05 a
Longmu 803	0.09 ± 0.05 a	0.24 ± 0.03 b
Salt Star	0.07 ± 0.03 a	0.25 ± 0.03 b
Spade	0.10 ± 0.06 a	0.44 ± 0.02 a
Gongnong No.1	0.07 ± 0.04 a	0.10 ± 0.09 a
Saiyuan	0.08 ± 0.02 a	0.32 ± 0.04 b
WL298 HQ	0.26 ± 0.03 a	0.54 ± 0.21 b
Knight 2	0.18 ± 0.06 a	0.38 ± 0.05 b
MF4020	0.14 ± 0.05 a	0.28 ± 0.02 b
Caoyuan No.2	0.09 ± 0.06 a	0.34 ± 0.03 b
Kangsai 2	0.05 ± 0.02 a	0.17 ± 0.03 b
Zhongmu No.1	0.06 ± 0.04 a	0.37 ± 0.11 b
Fudi	0.11 ± 0.05 a	0.23 ± 0.07 b
Challenger	0.09 ± 0.01 a	0.23 ± 0.04 b
Russia	0.06 ± 0.02 a	0.19 ± 0.08 b
Crown	0.08 ± 0.04 a	0.13 ± 0.08 a
Caoyuan No.3	0.12 ± 0.01 a	0.25 ± 0.13 a
Baimu 202	0.03 ± 0.03 a	0.10 ± 0.02 b
Juneng No.2	0.11 ± 0.05 a	0.26 ± 0.05 b
Juneng 201	0.16 ± 0.03 a	0.30 ± 0.05 b
Kateruo	0.11 ± 0.07 a	0.27 ± 0.08 b
Dryland	0.06 ± 0.03 a	0.23 ± 0.12 a
Gongnong No.2	0.28 ± 0.05 a	0.38 ± 0.02 b
Caoyuan No.1	0.24 ± 0.02 a	0.25 ± 0.07 a
Weiner	0.10 ± 0.03 a	0.28 ± 0.04 b
WL168 HQ	0.09 ± 0.04 a	0.21 ± 0.02 b
DY2006	0.07 ± 0.04 a	0.33 ± 0.06 b
Weiner	0.06 ± 0.02 a	0.11 ± 0.06 a
Dongnong No.1	0.08 ± 0.02 a	0.12 ± 0.05 a

Note: 4°C indicates cold treatment, while CK represents normal conditions.
