# Supplementary material for: Unveiling Salt Tolerance Mechanisms and Hub Genes in Alfalfa (Medicago sativa L.) Through Transcriptomic and WGCNA Analysis
Source: Plants (Basel). 2024 Nov 8;13(22):3141. doi: 10.3390/plants13223141 (PMC11597802; doi:10.3390/plants13223141)
Supplement: Supplementary file 1 [file plants-13-03141-s001.zip › Table S1~S13.zip!/Table S3.pdf]

Table S3. Effects of Cold Stress on the Fresh Weight of Different Alfalfa Varieties.

	Fresh Weight (g)			
	0d		10d	
	4°C	CK	4°C	CK
Juneng 401	0.0691 ± 0.0054 a	0.0699 ± 0.0040 a	0.0895 ± 0.0054 b	0.1091 ± 0.0084 c
Jinneng 5010	0.0579 ± 0.0049 a	0.0564 ± 0.0049 a	0.0692 ± 0.0049 a	0.1187 ± 0.0021 b
Juneng 551	0.0620 ± 0.0026 a	0.0643 ± 0.0042 a	0.0748 ± 0.0026 b	0.1056 ± 0.0070 c
Womu No. 1	0.0466 ± 0.0053 a	0.0464 ± 0.0107 a	0.0731 ± 0.0053 b	0.0801 ± 0.0068 c
Nongjing No. 1	0.0519 ± 0.0042 a	0.0537 ± 0.0032 a	0.0834 ± 0.0042 b	0.0839 ± 0.0108 c
SR4030	0.0496 ± 0.0027 a	0.0494 ± 0.0047 a	0.0787 ± 0.0027 b	0.6048 ± 0.4406 b
MF4015	0.0652 ± 0.0052 a	0.0655 ± 0.0041 a	0.0882 ± 0.0052 b	0.1420 ± 0.0144 c
Juneng No. 1	0.0655 ± 0.0051 a	0.0657 ± 0.0032 a	0.0717 ± 0.0051 a	0.0962 ± 0.0076 b
Instar	0.0703 ± 0.0041 a	0.0705 ± 0.0040 a	0.0724 ± 0.0041 a	0.0881 ± 0.0047 b
Longmu801	0.0775 ± 0.0012 a	0.0762 ± 0.0115 a	0.0879 ± 0.0012 a	0.0865 ± 0.0009 a
Golden Queen	0.0607 ± 0.0026 a	0.0622 ± 0.0071 a	0.0683 ± 0.0026 a	0.1033 ± 0.0174 a
Longmu 806	0.0590 ± 0.0168 a	0.0590 ± 0.0132 a	0.0841 ± 0.0168 b	0.0896 ± 0.0156 b
Zhaodong	0.0728 ± 0.0023 a	0.0726 ± 0.0022 a	0.1088 ± 0.0023 b	0.1507 ± 0.0053 c
Longmu 803	0.0569 ± 0.0013 a	0.0572 ± 0.0078 a	0.0664 ± 0.0013 a	0.1108 ± 0.0073 b
Salt Star	0.0770 ± 0.0161 a	0.0774 ± 0.0066 a	0.0998 ± 0.0161 ab	0.1095 ± 0.0156 b
Spade	0.0485 ± 0.0076 a	0.0487 ± 0.0045 a	0.1045 ± 0.0076 b	0.1340 ± 0.0051 c
Gongnong No.1	0.0491 ± 0.0093 a	0.0494 ± 0.0026 a	0.0976 ± 0.0093 b	0.1025 ± 0.0036 b
Saiyuan	0.0660 ± 0.0025 a	0.0660 ± 0.0119 a	0.0692 ± 0.0025 a	0.1197 ± 0.0048 b
WL298 HQ	0.0566 ± 0.0089 a	0.0569 ± 0.0104 a	0.0960 ± 0.0089 b	0.1347 ± 0.0028 c
Knight 2	0.0596 ± 0.0081 a	0.0594 ± 0.0042 a	0.0762 ± 0.0081 b	0.1008 ± 0.0082 c
MF4020	0.0628 ± 0.0074 a	0.0629 ± 0.0036 a	0.0774 ± 0.0074 b	0.1077 ± 0.0050 b
Caoyuan No.2	0.0440 ± 0.0041 a	0.0441 ± 0.0084 a	0.0699 ± 0.0041 b	0.1102 ± 0.0066 c
Kangsai 2	0.0682 ± 0.0023 a	0.0679 ± 0.0125 a	0.1052 ± 0.0023 b	0.1329 ± 0.0078 c
Zhongmu No.1	0.0840 ± 0.0077 a	0.0845 ± 0.0050 a	0.0968 ± 0.0077 ab	0.1119 ± 0.0035 c
Fudi	0.0818 ± 0.0054 a	0.0813 ± 0.0134 a	0.1231 ± 0.0054 b	0.1707 ± 0.0065 c
Challenger	0.0525 ± 0.0064 a	0.0524 ± 0.0069 a	0.0847 ± 0.0064 b	0.1264 ± 0.0046 c
Russia	0.0628 ± 0.0041 a	0.0631 ± 0.0035 a	0.0776 ± 0.0041 b	0.1341 ± 0.0038 c
Crown	0.0657 ± 0.0090 a	0.0662 ± 0.0018 a	0.0956 ± 0.0090 b	0.1133 ± 0.0074 b
Caoyuan No.3	0.0642 ± 0.0053 a	0.0639 ± 0.0061 a	0.0755 ± 0.0053 ab	0.0783 ± 0.0037 b
Baimu 202	0.0450 ± 0.0016 a	0.0455 ± 0.0060 a	0.0515 ± 0.0016 a	0.0759 ± 0.0049 b
Juneng No.2	0.0390 ± 0.0076 a	0.0386 ± 0.0040 a	0.0737 ± 0.0076 b	0.0752 ± 0.0072 b
Juneng 201	0.0557 ± 0.0124 a	0.0553 ± 0.0019 a	0.0958 ± 0.0124 b	0.1227 ± 0.0130 c
Kateruo	0.0550 ± 0.0040 a	0.0555 ± 0.0043 a	0.0588 ± 0.0040 b	0.0924 ± 0.0106 c
Dryland	0.0688 ± 0.0063 a	0.0683 ± 0.0025 a	0.0919 ± 0.0063 b	0.1112 ± 0.0072 c
Gongnong No.2	0.0698 ± 0.0076 a	0.0702 ± 0.0087 a	0.0920 ± 0.0076 b	0.1079 ± 0.0053 b
Caoyuan No.1	0.0572 ± 0.0014 a	0.0578 ± 0.0024 a	0.0687 ± 0.0014 b	0.0794 ± 0.0006 c
Weiner	0.0650 ± 0.0048 a	0.0653 ± 0.0070 a	0.0682 ± 0.0048 a	0.0920 ± 0.0022 b
WL168 HQ	0.0912 ± 0.0011 a	0.0916 ± 0.0036 a	0.1015 ± 0.0011 b	0.1064 ± 0.0028 b
DY2006	0.0746 ± 0.0054 a	0.0750 ± 0.0049 a	0.0839 ± 0.0054 b	0.0862 ± 0.0034 b
Weiner	0.0633 ± 0.0041 a	0.0635 ± 0.0070 a	0.0834 ± 0.0041 b	0.0841 ± 0.0052 b
Dongnong No.1	0.0768 ± 0.0055 a	0.0764 ± 0.0033 a	0.0972 ± 0.0055 b	0.0985 ± 0.0039 b

Note: 4°C indicates cold treatment, while CK represents normal conditions.
