# Supplementary material for: Unveiling Salt Tolerance Mechanisms and Hub Genes in Alfalfa (Medicago sativa L.) Through Transcriptomic and WGCNA Analysis
Source: Plants (Basel). 2024 Nov 8;13(22):3141. doi: 10.3390/plants13223141 (PMC11597802; doi:10.3390/plants13223141)
Supplement: Supplementary file 1 [file plants-13-03141-s001.zip › Table S1~S13.zip!/Table S4.pdf]

Table S4. Effects of Cold Stress on the Increase in Biomass of Different Alfalfa Varieties.

	Increase in Biomass (g)	
	0-10d	
	4°C	CK
Juneng 401	0.0205 ± 0.0013 a	0.0392 ± 0.0087 b
Jinneng 5010	0.0113 ± 0.0056 a	0.0623 ± 0.0067 b
Juneng 551	0.0128 ± 0.0047 a	0.0413 ± 0.0112 b
Womu No. 1	0.0264 ± 0.0153 a	0.0337 ± 0.0047 a
Nongjing No. 1	0.0315 ± 0.0086 a	0.0301 ± 0.0076 a
SR4030	0.0291 ± 0.0052 a	0.0400 ± 0.0031 b
MF4015	0.0229 ± 0.0062 a	0.0765 ± 0.0130 b
Juneng No. 1	0.0062 ± 0.0017 a	0.0305 ± 0.0058 a
Instar	0.0021 ± 0.0011 a	0.0175 ± 0.0086 b
Longmu801	0.0104 ± 0.0110 a	0.0104 ± 0.0112 a
Golden Queen	0.0076 ± 0.0081 a	0.0411 ± 0.0242 b
Longmu 806	0.0251 ± 0.0216 a	0.0307 ± 0.0285 a
Zhaodong	0.0360 ± 0.0041 a	0.0781 ± 0.0074 b
Longmu 803	0.0096 ± 0.0111 a	0.0536 ± 0.0059 b
Salt Star	0.0228 ± 0.0116 a	0.0321 ± 0.0120 a
Spade	0.0560 ± 0.0059 a	0.0853 ± 0.0043 b
Gongnong No.1	0.0485 ± 0.0142 a	0.0531 ± 0.0061 a
Saiyuan	0.0033 ± 0.0137 a	0.0537 ± 0.0162 b
WL298 HQ	0.0394 ± 0.0238 a	0.0777 ± 0.0109 a
Knight 2	0.0166 ± 0.0088 a	0.0414 ± 0.0055 b
MF4020	0.0146 ± 0.0082 a	0.0448 ± 0.0041 b
Caoyuan No.2	0.0259 ± 0.0090 a	0.0661 ± 0.0036 b
Kangsai 2	0.0370 ± 0.0148 a	0.0650 ± 0.0058 b
Zhongmu No.1	0.0128 ± 0.0133 a	0.0274 ± 0.0026 a
Fudi	0.0412 ± 0.0208 a	0.0893 ± 0.0069 b
Challenger	0.0322 ± 0.0100 a	0.0740 ± 0.0114 b
Russia	0.0148 ± 0.0073 a	0.0710 ± 0.0046 b
Crown	0.0299 ± 0.0076 a	0.0471 ± 0.0073 b
Caoyuan No.3	0.0113 ± 0.0040 a	0.0144 ± 0.0027 a
Baimu 202	0.0065 ± 0.0030 a	0.0304 ± 0.0109 b
Juneng No.2	0.0346 ± 0.0151 a	0.0366 ± 0.0111 a
Juneng 201	0.0401 ± 0.0119 a	0.0674 ± 0.0149 a
Kateruo	0.0038 ± 0.0062 a	0.0369 ± 0.0074 b
Dryland	0.0231 ± 0.0039 a	0.0428 ± 0.0094 b
Gongnong No.2	0.0221 ± 0.0101 a	0.0377 ± 0.0037 a
Caoyuan No.1	0.0115 ± 0.0054 a	0.0216 ± 0.0027 b
Weiner	0.0032 ± 0.0028 a	0.0267 ± 0.0076 b
WL168 HQ	0.0103 ± 0.0048 a	0.0148 ± 0.0025 a
DY2006	0.0093 ± 0.0030 a	0.0111 ± 0.0023 a
Weiner	0.0201 ± 0.0118 a	0.0206 ± 0.0059 a
Dongnong No.1	0.0203 ± 0.0045 a	0.0221 ± 0.0023 a

Note: 4°C indicates cold treatment, while CK represents normal conditions.
