# Supplementary material for: Unveiling Salt Tolerance Mechanisms and Hub Genes in Alfalfa (Medicago sativa L.) Through Transcriptomic and WGCNA Analysis
Source: Plants (Basel). 2024 Nov 8;13(22):3141. doi: 10.3390/plants13223141 (PMC11597802; doi:10.3390/plants13223141)
Supplement: Supplementary file 1 [file plants-13-03141-s001.zip › Table S1~S13.zip!/Table S5.pdf]

Table S5. Effects of Cold Stress on the Dry Weight of Different Alfalfa Varieties.

	Dry Weight (g)			
	0d		10d	
	4°C	ck	4°C	ck
Juneng 401	0.0156 ± 0.0024 a	0.0157 ± 0.0027 a	0.0104 ± 0.0017 a	0.0255 ± 0.0038 b
Jinneng 5010	0.0137 ± 0.0027 a	0.0135 ± 0.0027 a	0.0112 ± 0.0031 a	0.0211 ± 0.0010 c
Juneng 551	0.0162 ± 0.0018 b	0.0164 ± 0.0007 b	0.0129 ± 0.0003 a	0.0213 ± 0.0024 c
Womu No. 1	0.0112 ± 0.0012 a	0.0108 ± 0.0012 a	0.0108 ± 0.0007 a	0.0139 ± 0.0011 b
Nongjing No. 1	0.0082 ± 0.0017 a	0.0084 ± 0.0008 a	0.0150 ± 0.0032 b	0.0182 ± 0.0007 c
SR4030	0.0106 ± 0.0003 a	0.0105 ± 0.0012 a	0.0138 ± 0.0013 b	0.0134 ± 0.0009 b
MF4015	0.0126 ± 0.0009 a	0.0121 ± 0.0022 a	0.0164 ± 0.0020 b	0.0220 ± 0.0008 c
Juneng No. 1	0.0199 ± 0.0032 a	0.0198 ± 0.0038 a	0.0166 ± 0.0009 a	0.0230 ± 0.0029 a
Instar	0.0152 ± 0.0024 b	0.0157 ± 0.0016 b	0.0125 ± 0.0013 a	0.0189 ± 0.0037 bc
Longmu801	0.0206 ± 0.0010 b	0.0205 ± 0.0005 b	0.0135 ± 0.0015 a	0.0209 ± 0.0015 c
Golden Queen	0.0146 ± 0.0025 a	0.0145 ± 0.0019 a	0.0138 ± 0.0015 a	0.0175 ± 0.0012 b
Longmu 806	0.0117 ± 0.0028 a	0.0115 ± 0.0016 a	0.0144 ± 0.0030 a	0.0150 ± 0.0024 a
Zhaodong	0.0159 ± 0.0017 a	0.0159 ± 0.0019 a	0.0173 ± 0.0026 a	0.0282 ± 0.0024 b
Longmu 803	0.0108 ± 0.0018 a	0.0107 ± 0.0021 a	0.0142 ± 0.0019 ab	0.0240 ± 0.0023 c
Salt Star	0.0174 ± 0.0026 a	0.0179 ± 0.0012 a	0.0188 ± 0.0035 a	0.0190 ± 0.0034 a
Spade	0.0132 ± 0.0007 a	0.0135 ± 0.0009 a	0.0205 ± 0.0034 b	0.0230 ± 0.0016 b
Gongnong No.1	0.0123 ± 0.0006 a	0.0126 ± 0.0010 a	0.0215 ± 0.0007 b	0.0219 ± 0.0043 b
Saiyuan	0.0146 ± 0.0023 a	0.0148 ± 0.0033 a	0.0125 ± 0.0015 a	0.0192 ± 0.0032 b
WL298 HQ	0.0134 ± 0.0053 a	0.0130 ± 0.0028 a	0.0174 ± 0.0011 a	0.0215 ± 0.0018 b
Knight 2	0.0141 ± 0.0005 a	0.0146 ± 0.0029 a	0.0180 ± 0.0005 a	0.0203 ± 0.0040 a
MF4020	0.0119 ± 0.0013 a	0.0123 ± 0.0015 a	0.0139 ± 0.0014 a	0.0142 ± 0.0011 a
Caoyuan No.2	0.0094 ± 0.0027 a	0.0098 ± 0.0015 a	0.0155 ± 0.0014 a	0.0160 ± 0.0035 a
Kangsai 2	0.0170 ± 0.0053 a	0.0171 ± 0.0034 a	0.0214 ± 0.0003 b	0.0286 ± 0.0026 c
Zhongmu No.1	0.0159 ± 0.0021 a	0.0159 ± 0.0027 a	0.0169 ± 0.0008 a	0.0239 ± 0.0023 b
Fudi	0.0159 ± 0.0033 a	0.0159 ± 0.0015 a	0.0240 ± 0.0018 b	0.0324 ± 0.0065 c
Challenger	0.0124 ± 0.0012 a	0.0128 ± 0.0014 a	0.0170 ± 0.0006 b	0.0194 ± 0.0010 c
Russia	0.0143 ± 0.0013 a	0.0143 ± 0.0019 a	0.0153 ± 0.0009 a	0.0186 ± 0.0041 ab
Crown	0.0155 ± 0.0009 a	0.0153 ± 0.0008 a	0.0173 ± 0.0020 ab	0.0203 ± 0.0010 b
Caoyuan No.3	0.0130 ± 0.0005 a	0.0130 ± 0.0013 a	0.0152 ± 0.0013 a	0.0189 ± 0.0024 ab
Baimu 202	0.0104 ± 0.0008 a	0.0104 ± 0.0019 a	0.0134 ± 0.0009 b	0.0179 ± 0.0043 b
Juneng No.2	0.0094 ± 0.0031 a	0.0093 ± 0.0010 a	0.0202 ± 0.0004 b	0.0199 ± 0.0014 b
Juneng 201	0.0131 ± 0.0011 a	0.0134 ± 0.0010 a	0.0240 ± 0.0019 b	0.0253 ± 0.0050 b
Kateruo	0.0156 ± 0.0047 a	0.0162 ± 0.0053 a	0.0133 ± 0.0025 a	0.0221 ± 0.0034 ab
Dryland	0.0166 ± 0.0021 a	0.0166 ± 0.0010 a	0.0176 ± 0.0012 a	0.0293 ± 0.0038 b
Gongnong No.2	0.0172 ± 0.0027 a	0.0177 ± 0.0031 a	0.0203 ± 0.0010 a	0.0202 ± 0.0016 a
Caoyuan No.1	0.0129 ± 0.0013 a	0.0127 ± 0.0008 a	0.0141 ± 0.0042 a	0.0196 ± 0.0018 a
Weiner	0.0156 ± 0.0004 b	0.0155 ± 0.0021 b	0.0081 ± 0.0003 a	0.0250 ± 0.0008 c
WL168 HQ	0.0196 ± 0.0025 a	0.0195 ± 0.0023 a	0.0191 ± 0.0010 a	0.0238 ± 0.0058 a
DY2006	0.0153 ± 0.0031 a	0.0156 ± 0.0011 a	0.0168 ± 0.0027 a	0.0246 ± 0.0025 b
Weiner	0.0164 ± 0.0029 a	0.0167 ± 0.0017 a	0.0178 ± 0.0005 a	0.0211 ± 0.0010 b
Dongnong No.1	0.0151 ± 0.0038 a	0.0154 ± 0.0034 a	0.0172 ± 0.0015 a	0.0193 ± 0.0035 a

Note: 4°C indicates cold treatment, while CK represents normal conditions.
