# Supplementary material for: Unveiling Salt Tolerance Mechanisms and Hub Genes in Alfalfa (Medicago sativa L.) Through Transcriptomic and WGCNA Analysis
Source: Plants (Basel). 2024 Nov 8;13(22):3141. doi: 10.3390/plants13223141 (PMC11597802; doi:10.3390/plants13223141)
Supplement: Supplementary file 1 [file plants-13-03141-s001.zip › Table S1~S13.zip!/Table S6.pdf]

Table S6. Effects of Cold Stress on Relative Growth Indicators of Different Alfalfa Varieties.

	Relative Plant Height	Relative Growth Rate	Relative Fresh Weight	Relative Increase in Biomass	Relative Dry Weight
Juneng 401	0.85 ± 0.03	0.58 ± 0.16	0.8243 ± 0.0848	0.5343 ± 0.0805	0.4131 ± 0.0761
Jinneng 5010	0.79 ± 0.05	0.12 ± 0.08	0.5833 ± 0.0415	0.1782 ± 0.0717	0.5293 ± 0.1417
Juneng 551	0.75 ± 0.01	0.39 ± 0.08	0.7117 ± 0.0702	0.3288 ± 0.1751	0.6117 ± 0.0716
Womu No. 1	0.69 ± 0.07	0.31 ± 0.15	0.9196 ± 0.1438	0.7609 ± 0.3877	0.7805 ± 0.1106
Nongjing No. 1	0.90 ± 0.03	0.76 ± 0.10	1.0015 ± 0.0863	1.0581 ± 0.2619	0.8292 ± 0.2058
SR4030	0.74 ± 0.04	0.26 ± 0.01	0.8826 ± 0.0571	0.7294 ± 0.1326	1.0317 ± 0.0585
MF4015	0.76 ± 0.09	0.08 ± 0.08	0.6280 ± 0.1042	0.3147 ± 0.1396	0.7423 ± 0.0793
Juneng No. 1	0.94 ± 0.07	0.64 ± 0.19	0.7472 ± 0.0591	0.2090 ± 0.0762	0.7329 ± 0.1226
Instar	0.89 ± 0.05	0.35 ± 0.15	0.8248 ± 0.0892	0.1464 ± 0.1204	0.6749 ± 0.1393
Longmu801	0.92 ± 0.03	0.50 ± 0.29	1.0154 ± 0.0043	1.0130 ± 0.0303	0.6476 ± 0.0727
Golden Queen	0.86 ± 0.05	0.78 ± 0.76	0.6728 ± 0.1036	0.1196 ± 0.1925	0.7898 ± 0.1251
Longmu 806	0.92 ± 0.02	0.98 ± 0.53	0.9431 ± 0.1489	0.8920 ± 0.1176	0.9716 ± 0.2102
Zhaodong	0.99 ± 0.01	0.93 ± 0.14	0.7226 ± 0.0107	0.4605 ± 0.0154	0.6205 ± 0.1439
Longmu 803	0.87 ± 0.06	0.38 ± 0.23	0.6017 ± 0.0527	0.1725 ± 0.2119	0.5991 ± 0.1288
Salt Star	0.79 ± 0.03	0.30 ± 0.14	0.9097 ± 0.0476	0.6931 ± 0.2025	0.9907 ± 0.0975
Spade	0.69 ± 0.07	0.22 ± 0.12	0.7790 ± 0.0282	0.6557 ± 0.0590	0.8862 ± 0.0837
Gongnong No.1	0.95 ± 0.04	0.87 ± 0.23	0.9509 ± 0.0571	0.9025 ± 0.1587	1.0067 ± 0.1845
Saiyuan	0.77 ± 0.04	0.24 ± 0.05	0.5795 ± 0.0422	0.0071 ± 0.2805	0.6714 ± 0.1779
WL298 HQ	0.75 ± 0.12	0.54 ± 0.21	0.7135 ± 0.0708	0.4833 ± 0.2547	0.8132 ± 0.0193
Knight 2	0.84 ± 0.04	0.46 ± 0.09	0.7634 ± 0.1430	0.4186 ± 0.2523	0.9122 ± 0.1855
MF4020	0.88 ± 0.04	0.53 ± 0.22	0.7213 ± 0.0889	0.3372 ± 0.1986	0.9773 ± 0.0777
Caoyuan No.2	0.81 ± 0.03	0.29 ± 0.18	0.6359 ± 0.0575	0.3876 ± 0.1194	0.6985 ± 0.5923
Kangsai 2	0.90 ± 0.01	0.32 ± 0.15	0.7926 ± 0.0347	0.5607 ± 0.1767	0.7514 ± 0.0685
Zhongmu No.1	0.75 ± 0.06	0.14 ± 0.09	0.8659 ± 0.0765	0.4396 ± 0.4687	0.7133 ± 0.0873
Fudi	0.91 ± 0.06	0.49 ± 0.18	0.7226 ± 0.0590	0.4512 ± 0.2019	0.7666 ± 0.2014

Challenger	0.86 ± 0.04	0.42 ± 0.12	0.6698 ± 0.0481	0.4298 ± 0.0972	0.8805 ± 0.0767
Russia	0.87 ± 0.09	0.33 ± 0.16	0.5786 ± 0.0184	0.2051 ± 0.0891	0.8507 ± 0.1914
Crown	0.94 ± 0.03	0.65 ± 0.06	0.8439 ± 0.0555	0.6298 ± 0.0976	0.8506 ± 0.0634
Caoyuan No.3	0.88 ± 0.11	0.59 ± 0.33	0.9636 ± 0.0225	0.8286 ± 0.3927	0.8100 ± 0.0892
Baimu 202	0.91 ± 0.03	0.35 ± 0.37	0.6812 ± 0.0593	0.2087 ± 0.0466	0.7697 ± 0.1290
Juneng No.2	0.86 ± 0.05	0.44 ± 0.16	0.9799 ± 0.0275	0.9208 ± 0.1452	1.0205 ± 0.0817
Juneng 201	0.84 ± 0.09	0.56 ± 0.20	0.7929 ± 0.1808	0.6409 ± 0.3166	0.9643 ± 0.1195
Kateruo	0.85 ± 0.10	0.47 ± 0.41	0.6390 ± 0.0441	0.1128 ± 0.1657	0.6205 ± 0.2087
Dryland	0.84 ± 0.13	0.32 ± 0.18	0.8318 ± 0.1102	0.5672 ± 0.2140	0.6091 ± 0.1052
Gongnong No.2	0.92 ± 0.04	0.74 ± 0.16	0.8517 ± 0.0330	0.5738 ± 0.2279	1.0050 ± 0.0332
Caoyuan No.1	1.00 ± 0.04	1.02 ± 0.26	0.8653 ± 0.0221	0.5182 ± 0.1954	0.7186 ± 0.1897
Weiner	0.81 ± 0.01	0.35 ± 0.06	0.7425 ± 0.0593	0.1224 ± 0.0944	0.3252 ± 0.0168
WL168 HQ	0.85 ± 0.05	0.43 ± 0.19	0.9542 ± 0.0326	0.7200 ± 0.3961	0.8270 ± 0.1585
DY2006	0.76 ± 0.02	0.21 ± 0.10	0.9731 ± 0.0262	0.8426 ± 0.2882	0.6964 ± 0.1850
Weiner	0.95 ± 0.05	0.65 ± 0.15	0.9935 ± 0.0557	0.9164 ± 0.3610	0.8428 ± 0.0166
Dongnong No.1	0.94 ± 0.04	0.68 ± 0.15	0.9866 ± 0.0307	0.9147 ± 0.1130	0.9198 ± 0.2567
