# Supplementary material for: Unveiling Salt Tolerance Mechanisms and Hub Genes in Alfalfa (Medicago sativa L.) Through Transcriptomic and WGCNA Analysis
Source: Plants (Basel). 2024 Nov 8;13(22):3141. doi: 10.3390/plants13223141 (PMC11597802; doi:10.3390/plants13223141)
Supplement: Supplementary file 1 [file plants-13-03141-s001.zip › Table S1~S13.zip!/Table S7.pdf]

Table S7. Unweighted and Weighted Grey Correlation of Different Alfalfa Varieties.

	Unweighted Correlation	Weighted Correlation
Juneng 401	0.5820	0.5457
Jinneng 5010	0.4923	0.4451
Juneng 551	0.5316	0.4944
Womu No. 1	0.6112	0.5943
Nongjing No. 1	0.7855	0.7888
SR4030	0.6574	0.6277
MF4015	0.5194	0.4767
Juneng No. 1	0.6109	0.5467
Instar	0.5722	0.5070
Longmu801	0.7289	0.7152
Golden Queen	0.5994	0.5513
Longmu 806	0.8053	0.7907
Zhaodong	0.6715	0.6225
Longmu 803	0.5327	0.4783
Salt Star	0.6586	0.6218
Spade	0.5861	0.5620
Gongnong No.1	0.8041	0.7777
Saiyuan	0.5005	0.4511
WL298 HQ	0.5800	0.5496
Knight 2	0.6101	0.5622
MF4020	0.6288	0.5727
Caoyuan No.2	0.5978	0.5489
Kangsai 2	0.6063	0.5551
Zhongmu No.1	0.5590	0.5171
Fudi	0.6025	0.5499
Challenger	0.5959	0.5469
Russia	0.5628	0.5047
Crown	0.6768	0.6267
Caoyuan No.3	0.6947	0.6660
Baimu 202	0.5722	0.5087
Juneng No.2	0.7430	0.7177
Juneng 201	0.6561	0.6195
Kateruo	0.5377	0.4850
Dryland	0.5808	0.5394
Gongnong No.2	0.7081	0.6586
Caoyuan No.1	0.7350	0.6879
Weiner	0.5092	0.4602
WL168 HQ	0.6599	0.6218
DY2006	0.6303	0.6117
Weiner	0.7499	0.7192
Dongnong No.1	0.7657	0.7353
