# Supplementary material for: Unveiling Salt Tolerance Mechanisms and Hub Genes in Alfalfa (Medicago sativa L.) Through Transcriptomic and WGCNA Analysis
Source: Plants (Basel). 2024 Nov 8;13(22):3141. doi: 10.3390/plants13223141 (PMC11597802; doi:10.3390/plants13223141)
Supplement: Supplementary file 1 [file plants-13-03141-s001.zip › Table S1~S13.zip!/Table S8.pdf]

Table S8. Statistical table for assessment of sample sequencing data.

Sample	Raw Data		Valid Data		Valid Ratio%	Q20%	Q30%	GC content%
	Read	Base	Read	Base				
LM_CK1	37538822	5.63G	35930510	5.39G	95.72	99.20	96.85	42
LM_CK2	41435486	6.22G	39775910	5.97G	95.99	99.24	96.97	42
LM_CK3	43229200	6.48G	41592130	6.24G	96.21	99.31	97.05	41.50
LM_CK4	40606088	6.09G	39352780	5.90G	96.91	99.44	97.47	41.50
LM_ST1	39181810	5.88G	37845170	5.68G	96.59	99.36	97.35	42.50
LM_ST2	39220438	5.88G	37859570	5.68G	96.53	99.39	97.36	42.50
LM_ST3	42998856	6.45G	41421292	6.21G	96.33	99.37	97.08	42.50
LM_ST4	40473314	6.07G	39128456	5.87G	96.68	99.41	97.50	42
WL_CK1	38161626	5.72G	36722816	5.51G	96.23	99.38	97.39	41.50
WL_CK2	37450844	5.62G	35987452	5.40G	96.09	99.40	97.33	41.50
WL_CK3	40379350	6.06G	38263098	5.74G	94.76	99.36	97.32	41.50
WL_CK4	37655692	5.65G	35845360	5.38G	95.19	99.37	97.27	41.50
WL_ST1	40701480	6.11G	38708232	5.81G	95.10	99.22	96.59	41.50
WL_ST2	36959530	5.54G	34829836	5.22G	94.24	99.23	96.76	41.50
WL_ST3	37291486	5.59G	35441784	5.32G	95.04	99.26	96.65	41.50
WL_ST4	35512472	5.33G	33821714	5.07G	95.24	99.38	97.28	42.50
