# Supplementary material for: Unveiling Salt Tolerance Mechanisms and Hub Genes in Alfalfa (Medicago sativa L.) Through Transcriptomic and WGCNA Analysis
Source: Plants (Basel). 2024 Nov 8;13(22):3141. doi: 10.3390/plants13223141 (PMC11597802; doi:10.3390/plants13223141)
Supplement: Supplementary file 1 [file plants-13-03141-s001.zip › Table S1~S13.zip!/Table S9.pdf]

Table S9. Comparative Analysis of Different Alfalfa Genomes.

Sample	Valid reads	Mapped reads (Zhongmu NO.4)	Mapped reads (Xinjiangdaye)	Mapped reads (Zhongmu NO.1)
LM_CK1	35930510	84.21%	84.06%	69.02%
LM_CK2	39775910	86.11%	85.96%	70.51%
LM_CK3	41592130	87.29%	87.13%	71.52%
LM_CK4	39352780	89.91%	89.75%	73.67%
LM_ST1	37845170	85.43%	85.32%	69.43%
LM_ST2	37859570	86.09%	85.98%	69.94%
LM_ST3	41421292	86.08%	85.95%	69.92%
LM_ST4	39128456	86.32%	86.23%	70.08%
WL_CK1	36722816	86.24%	85.84%	70.62%
WL_CK2	35987452	88.97%	88.55%	72.85%
WL_CK3	38263098	89.94%	89.52%	73.51%
WL_CK4	35845360	89.77%	89.33%	73.49%
WL_ST1	38708232	88.87%	88.50%	72.25%
WL_ST2	39500234	88.82%	88.69%	72.39%
WL_ST3	35441784	89.33%	88.95%	72.52%
WL_ST4	37253832	84.75%	84.96%	69.41%
Average	38164289	87.38%	87.17%	71.32%
